# PgaB orthologues contain a glycoside hydrolase domain that cleaves deacetylated poly-β(1,6)-*N*-acetylglucosamine and can disrupt bacterial biofilms

**DOI:** 10.1371/journal.ppat.1006998

**Published:** 2018-04-23

**Authors:** Dustin J. Little, Roland Pfoh, François Le Mauff, Natalie C. Bamford, Christina Notte, Perrin Baker, Manita Guragain, Howard Robinson, Gerald B. Pier, Mark Nitz, Rajendar Deora, Donald C. Sheppard, P. Lynne Howell

**Affiliations:** 1 Program in Molecular Medicine, The Hospital for Sick Children, Toronto, ON, Canada; 2 Department of Biochemistry, University of Toronto, Toronto, ON, Canada; 3 Departments of Medicine and of Microbiology and Immunology, McGill University, Montréal, QC, Canada; 4 Infectious Diseases and Immunity in Global Health Program, Research Institute of the McGill University Health Centre, Montréal, QC, Canada; 5 Department of Microbiology and Immunology, Wake Forest School of Medicine, Winston-Salem, NC, United States of America; 6 Department of Microbial Infection and Immunity, The Ohio State University Wexner Medical Center, Columbus, OH, United States of America; 7 Photon Sciences Division, Brookhaven National Laboratory, Upton, NY, United States of America; 8 Division of Infectious Diseases, Department of Medicine, Brigham and Women's Hospital, Harvard Medical School, Boston, MA, United States of America; 9 Department of Chemistry, University of Toronto, Toronto, ON, Canada; Channing Laboratory, Brigham and Women's Hospital, UNITED STATES

## Abstract

Poly-β(1,6)-*N*-acetyl-D-glucosamine (PNAG) is a major biofilm component of many pathogenic bacteria. The production, modification, and export of PNAG in *Escherichia coli* and *Bordetella* species require the protein products encoded by the *pgaABCD* operon. PgaB is a two-domain periplasmic protein that contains an N-terminal deacetylase domain and a C-terminal PNAG binding domain that is critical for export. However, the exact function of the PgaB C-terminal domain remains unclear. Herein, we show that the C-terminal domains of *Bordetella bronchiseptica* PgaB (PgaB_*Bb*_) and *E*. *coli* PgaB (PgaB_*Ec*_) function as glycoside hydrolases. These enzymes hydrolyze purified deacetylated PNAG (dPNAG) from *Staphylococcus aureus*, disrupt PNAG-dependent biofilms formed by *Bordetella pertussis*, *Staphylococcus carnosus*, *Staphylococcus epidermidis*, and *E*. *coli*, and potentiate bacterial killing by gentamicin. Furthermore, we found that PgaB_*Bb*_ was only able to hydrolyze PNAG produced *in situ* by the *E*. *coli* PgaCD synthase complex when an active deacetylase domain was present. Mass spectrometry analysis of the PgaB-hydrolyzed dPNAG substrate showed a GlcN-GlcNAc-GlcNAc motif at the new reducing end of detected fragments. Our 1.76 Å structure of the C-terminal domain of PgaB_*Bb*_ reveals a central cavity within an elongated surface groove that appears ideally suited to recognize the GlcN-GlcNAc-GlcNAc motif. The structure, in conjunction with molecular modeling and site directed mutagenesis led to the identification of the dPNAG binding subsites and D474 as the probable catalytic acid. This work expands the role of PgaB within the PNAG biosynthesis machinery, defines a new glycoside hydrolase family GH153, and identifies PgaB as a possible therapeutic agent for treating PNAG-dependent biofilm infections.

## Author summary

From plaque on teeth to infections in the lungs of cystic fibrosis patients, biofilms are a serious health concern and difficult to eradicate. One of the key building blocks involved in biofilm formation are polymeric sugar compounds that are secreted by the bacteria. Our work focuses on the biopolymer poly-β(1,6)-*N*-acetyl-D-glucosamine (PNAG), which is produced by numerous pathogenic organisms. Deacetylation of PNAG by the N-terminal domain of PgaB is a critical step in polymer maturation and is required for the formation of robust biofilms. Herein, we show that the C-terminal domain of PgaB is a glycoside hydrolase active on partially deacetylated PNAG, and that the enzyme disrupts PNAG-dependent biofilms and potentiates killing by antibiotics. Only deacetylated PNAG could be cleaved, suggesting that PgaB deacetylates and hydrolyses the polymer in sequential order. Analyzing the chemical structure of the cleaved dPNAG fragments revealed a distinct motif of sugar units. Structural and functional studies identify key amino acids positioned in an elongated polymer-binding groove that potentially recognize the sugar motif during cleavage. Our study provides further insight into the mechanism of periplasmic PNAG modification, and suggests PgaB could be utilized as a therapeutic agent to eliminate biofilms.

## Introduction

A major determinant of *Bordetella* pathogenicity is their ability to form biofilms on biotic and abiotic surfaces [1–9]. *Bordetella pertussis* and *parapertussis* are the causative agents of whooping cough in humans, whereas *Bordetella bronchiseptica* has a broad host range, colonizing and causing respiratory diseases in a wide variety of animals including kennel cough in dogs [10, 11]. Biofilm formation by *B*. *bronchiseptica* requires production of poly-β(1,6)-*N*-acetyl-D-glucosamine (PNAG). PNAG produced by *B*. *bronchiseptica* was originally called *B**ordetella* polysaccharide, or Bps, and was shown to be important for innate immune resistance and colonization of the mouse respiratory tract [6, 7, 12, 13]. PNAG production, modification, and export in *Bordetella* species are dependent on the *bpsABCD* operon [12, 14]. This operon has recently been re-annotated in databases as *pgaABCD*. PNAG is an important virulence factor for infection by other bacteria such as *Escherichia coli* [15], *Staphylococcus aure*us [16], *Staphylococcus epidermidis* [17], and *Klebsiella pneumoniae* [18], and is also produced by numerous fungal and eukaryotic organisms including Plasmodia spp. which are the causative agent of malaria [19].

PNAG production in Gram-negative bacteria is initiated by the PgaC and PgaD synthase complex [20]. PgaC is an inner-membrane protein that contains a glycosyltransferase domain and interacts with the inner-membrane protein PgaD in a bis-(3’,5’)-cyclic-dimeric-guanosine monophosphate (c-di-GMP) dependent manner [21]. Together PgaCD synthesize and transport PNAG across the cytoplasmic membrane [21]. PgaA is a two-domain protein that contains an N-terminal periplasmic tetratricopeptide repeat (TPR) interaction domain and a C-terminal outer-membrane porin that facilitates PNAG export across the outer membrane [22]. The final protein is PgaB, a two-domain periplasmic protein with an N-terminal family four carbohydrate esterase (CE4) domain that displays metal-dependent deacetylation activity on PNAG oligomers [14, 23]. In *B*. *bronchiseptica* the formation of deacetylated PNAG (dPNAG) does not appear to be required for polymer transport through the outer-membrane, but is required for the formation of a robust three-dimensional biofilm [14]. The C-terminal domain of PgaB is annotated as a member of the glycoside hydrolase (GH) 13-like family. For the *E*. *coli* PgaB orthologue we previously hypothesized this domain may play a role in binding and translocating PNAG through the periplasmic space [24]. To date, there is no experimental evidence demonstrating hydrolytic activity for this putative GH domain. However, carbohydrate-cleaving enzymes have been identified and characterized in other exopolysaccharide (EPS) biosynthetic systems revealing important roles for their hydrolase activity in polymer production and/or biofilm formation. *E*. *coli* BcsZ is a periplasmic cellulase that has been shown to hydrolyze agar embedded carboxymethylcellulose and is required for efficient cellulose biosynthesis and export [25, 26]. *Listeria monocytogenes* PssZ is a glycoside hydrolase important for production of the *N*-acetylmannosamine-galactose rich EPS that is required for cell aggregation [27]. In alginate biosynthesis the periplasmic lyase AlgL has been implicated in polymer biosynthesis [28] and is proposed to be a part the alginate trans-envelope complex. Glycoside hydrolases have also been shown to play a role in fungal EPS biosynthesis, as galactosaminogalactan produced by *Aspergillus fumigatus* requires Sph3 [29]. Although the exact function of these GHs in polymer biosynthesis remains unclear, there exists a strong body of data that shows EPS biosynthetic systems frequently contain an active and specific carbohydrate-cleaving enzyme.

Herein, we demonstrate that the C-terminal domain of *B*. *bronchiseptica* PgaB (formerly called BpsB, but referred to as PgaB_*Bb*_ henceforth) and *E*. *coli* PgaB (PgaB_*Ec*_) are dPNAG hydrolases that can cleave purified or *in situ* produced dPNAG, disrupt pre-formed PNAG-dependent biofilms, and potentiate antibiotic killing by gentamicin. Furthermore, our structure-function analysis of PgaB suggests that deacetylated PNAG is the substrate for hydrolysis, identifies a GlcN-GlcNAc-GlcNAc motif required for cleavage of the polymer, and defines a new GH family.

## Results

### PgaB displays glycoside hydrolase activity and disrupts PNAG-dependent biofilms

**Fig 1:**
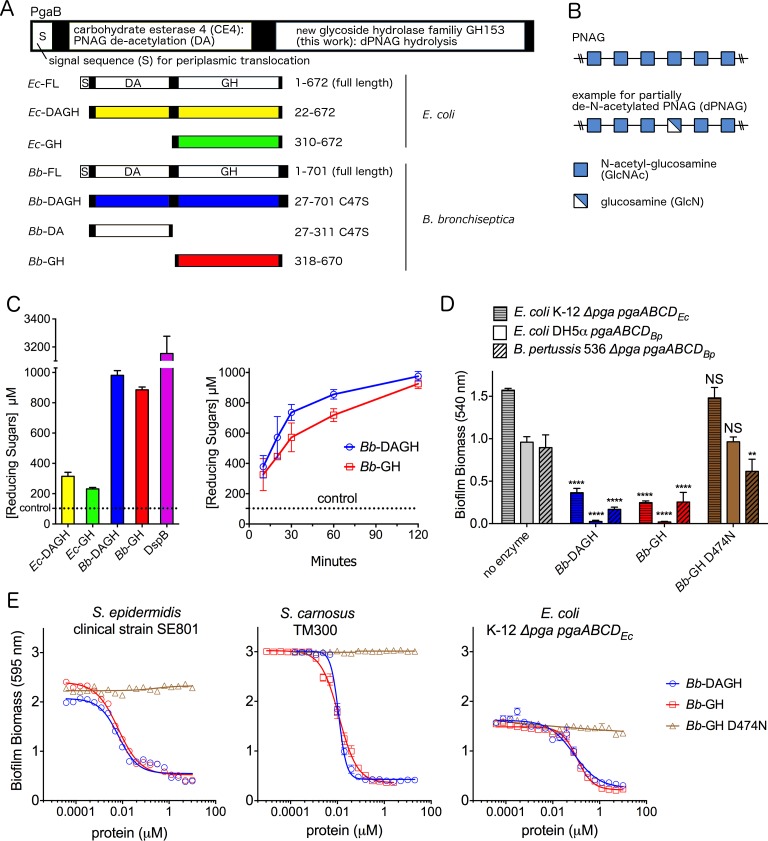
PgaB exhibits glycoside hydrolase activity. Schematic representation of the (A) PgaB constructs used in this study and (B) PNAG and dPNAG composition. (C) Reducing sugar assay with 2 mg/ml dPNAG purified from *S*. *aureus* with 2 μM PgaB variants and DspB over 24 h and PgaB_*Bb*_ hydrolase activity against dPNAG over 2 h. Error bars represent the standard error (S.E.) from two independent experiments performed in duplicate. (D) Biofilm disruption assay performed with 1.3 μM enzyme and the indicated strains. ****P ≤ 0.0001, **P ≤ 0.01, NS: no significant difference. Statistical significance was evaluated using two-way analysis of variance and Tukey’s multiple comparison test. (E) Dose response curves examining *S*. *carnosus*, *S*. *epidermidis*, and *E*. *coli* biofilm disruption. In panels D/E the error bars represent the S.E. and n = 3. In all graphs, *Bb*-DAGH is coloured blue, *Bb*-GH red, and the D474N variant brown.

Our previous studies using PNAG oligomers (up to a hexamer) or artificial *para-*nitrophenyl glycoside substrates and PgaB_*Ec*_ failed to demonstrate glycoside hydrolase activity [23, 24]. As we obtained similar results for PgaB_*Bb*_ [14], we hypothesized that PgaB may require longer, high molecular weight PNAG for GH activity to occur. Since a reducing sugar assay with purified exopolysaccharide had been successfully used previously to demonstrate GH activity for PslG and Sph3 [29, 30], we purified dPNAG (~5% deacetylated) from *S*. *aureus* and tested whether incubating this substrate with PgaB_*Ec*_ or PgaB_*Bb*_ resulted in an increase of soluble reducing sugars. We tested full-length constructs of PgaB that contain both the deacetylase (DA) and GH domain (*Ec*-DAGH and *Bb*-DAGH, which share 38% sequence identity), the isolated GH domains (*Ec*-GH and *Bb*-GH, which share 44% sequence identity), as well as the PNAG glycoside hydrolase dispersin B (DspB) (Fig 1). Incubation of purified dPNAG (Fig 1B) with 2 μM *Ec*-DAGH, *Ec*-GH, *Bb*-DAGH, *Bb*-GH, and DspB for 24 h resulted in an increase in concentration of soluble reducing sugars of 305 ± 19, 232 ± 7, 981 ± 22, 886 ± 13, and 3125 ± 124 μM respectively (Fig 1C). Given that the initial dPNAG substrate solution had 103 ± 10 μM of reducing sugars, this equates to a 3.0, 2.3, 9.5, 8.6 and 30.6 -fold increase in the number of reducing sugars for *Ec*-DAGH, *Ec*-GH, *Bb*-DAGH, *Bb*-GH, and DspB, respectively, over the course of the assay. As PgaB_*Bb*_ exhibited a higher level of GH activity than PgaB_*Ec*_, we monitored the reaction of PgaB_*Bb*_ over 2 h. The 2 h time-course showed an increase in reducing sugars that approached saturation with similar amounts of reducing sugar produced to that of the 24 h samples (Fig 1C).

DspB has been shown to disperse PNAG-dependent biofilms in *ex vivo* assays [12, 31–33]. Therefore, to further probe the activity of PgaB, we examined whether PgaB_*Bb*_ could disrupt pre-formed PNAG biofilms produced by *E*. *coli* and *B*. *pertussis* (Fig 1D). We utilized three different bacterial strains that have been engineered to over-produce PNAG from either the *pgaABCD*_*Ec*_ or *pgaABCD*_*Bb*_ operons [6, 34]. Incubating *Bb*-DAGH and *Bb*-GH with preformed *E*. *coli* or *B*. *pertussis* biofilms completely disrupted the biofilms within 2 h. To examine whether biofilm disruption was dependent on PgaB_*Bb*_ glycoside hydrolase activity and not the presence of the protein, we compared the sequence of PgaB_*Bb*_ to members of the GH13 family and identified D474 as the putative nucleophile required for hydrolysis. Thus, we constructed a D474N variant and examined its ability to disrupt preformed biofilms. The D474N mutant was unable to disrupt the PNAG biofilms produced by *E*. *coli* yet displayed a small, but significant (p <0.01), ability to disrupt the PNAG biofilm produced by *B*. *pertussis*. These data suggest that the observed reduction in biofilm biomass is a direct result of the catalytic activity of PgaB_*Bb*_.

*B*. *pertussis* is a slow growing bacterium that requires at least 72 h to produce a relatively small amount of biomass (OD_540_ levels of around 1). Therefore, to determine the effective concentration to disrupt 50% of the biofilm (EC_50_) for PgaB_*Bb*_ we utilized the faster growing *E*. *coli* K-12 and *S*. *carnosus* TM300 PNAG-overproducing strains, and a clinical *S*. *epidermidis* strain [35]. Treatment of the preformed biofilms for 2 h with *Bb*-DAGH and *Bb*-GH disrupted the biofilms with EC_50_ values in the nM range. The EC_50_ values for the *Bb*-DAGH and *Bb*-GH were 6.7 ± 0.9 and 6.4 ± 0.6 nM, respectively, for *S*. *epidermidis*; 11.4 ± 0.3 and 11.7 ± 0.6 nM, respectively, for *S*. *carnosus*; and 80.6 ± 14.9 and 108.1 ± 8.3 nM, respectively for *E*. *coli* (Fig 1E). Similar to the end-point assay, the D474N mutant showed little to no ability to disrupt the biofilm biomass. Collectively, these data suggest that the C-terminal domain of PgaB_*Ec*_ and PgaB_*Bb*_ exhibit dPNAG hydrolase activity and their catalytic function can disrupt biofilms produced from various biological sources.

### Disruption of PNAG-dependent biofilms by PgaB potentiates antibiotic killing

**Fig 2:**
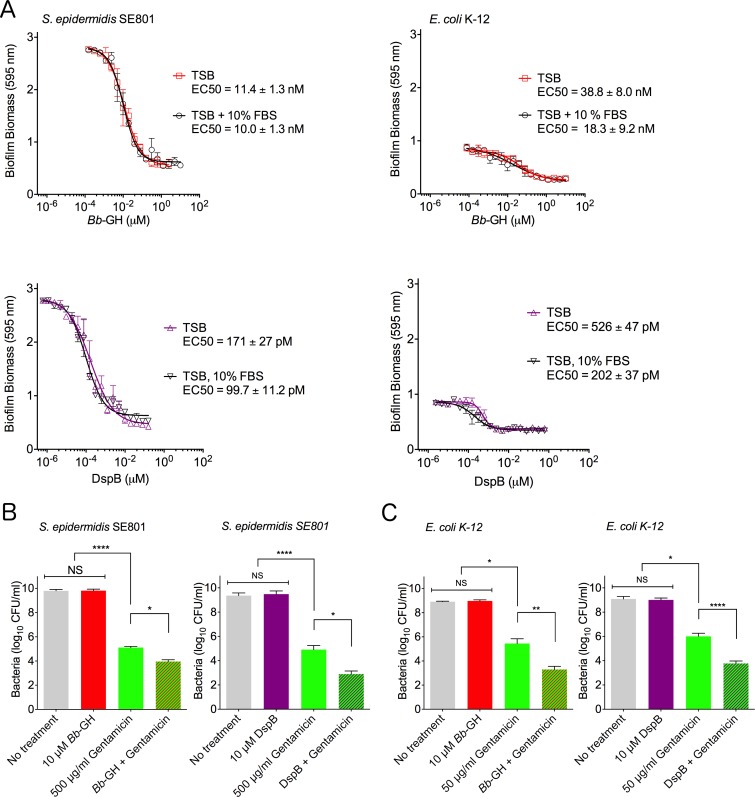
*Bb*-GH is active in fetal bovine serum and potentiates killing by gentamicin. (A) Dose response curves investigating the effect of fetal bovine serum on *Bb*-GH and DspB activity during disruption of *S*. *epidermidis* SE801 and *E*. *coli* K-12 biofilms. Error bars represent S.E., n = 3. (B) Enumeration of *S*. *epidermidis* SE801 after biofilm treatment with *Bb*-GH or DspB and 500 μg/ml gentamicin for 20 h. (C) Enumeration of *E*. *coli* K-12 after biofilm treatment with *Bb*-GH or DspB and 50 μg/ml gentamicin for 4 h. For (B) and (C) the mean was calculated from three independent experiments, error bars represent S.E.M. Statistical significance was calculated using one-way analysis of variance and Tukey’s multiple comparison test. ****P ≤ 0.0001, **P ≤ 0.01, *P ≤ 0.05, NS: no significant difference.

Bacteria form biofilms as a mechanism to evade the host immune response and to limit killing by antibiotics. To evaluate the potential of PgaB as a therapeutic agent, we tested the effect of serum on PgaB activity, examined whether PgaB could potentiate antibiotic killing, and compared these activities to DspB. Preformed biofilms of *S*. *epidermidis* and *E*. *coli* grown in the presence of TSB media were treated with *Bb*-GH or DspB in the presence and absence of fetal bovine serum and EC_50_ values determined. Serum had a minimal effect on the disruption activity of *Bb*-GH with an observed EC_50_ = 11.4 ± 1.3 nM (without serum) and 10.0 ± 1.3 nM (with serum) for *S*. *epidermidis* biofilms; and EC_50_ values of 38.8 ± 8.0 nM (without serum) and 18.3 ± 9.2 nM (with serum) for *E*. *coli* biofilms (Fig 2A). DspB displayed 60–100 times higher activity compared to *Bb*-GH with EC_50_ values of 171 ± 27 pM (without serum) and 99.7 ± 11.2 pM (with serum), and 526 ± 47 pM (without serum) and 202 ± 37 pM (with serum) for the *S*. *epidermidis* and *E*. *coli* biofilms, respectively (Fig 2A). Next, preformed *S*. *epidermidis* and *E*. *coli* biofilms were incubated with gentamicin alone or in combination with either *Bb*-GH or DspB, and bacteria were enumerated after 20 and 4 h treatments, respectively (Fig 2B). Gentamicin was chosen for these experiments as vancomycin has been shown previously to have minimal bactericidal effect on non-replicating cells [36], and it displays bactericidal activity against Gram-positive and -negative bacteria. Biofilm-embedded *S*. *epidermidis* showed a modest but significant 1-log reduction in cfu/ml in the presence of 500 μg/ml gentamicin and *Bb*-GH, versus antibiotic alone (Fig 2B). The enzyme was more effective at potentiating bacteria killing for biofilm-embedded *E*. *coli*, as there was a greater than 2-log reduction in cfu/ml in the presence of 50 μg/ml gentamicin and *Bb*-GH relative to antibiotic treatment alone (Fig 2C). Biofilm treatment with the hydrolase alone had no effect on the bacteria. In comparison, DspB displayed a 2-log reduction versus gentamicin alone for both *S*. *epidermidis* and *E*. *coli*. Together these results further support the potential of PgaB as an antimicrobial agent albeit it has a higher EC_50_ value than DspB for biofilm dispersal.

### PgaB glycoside hydrolase activity requires deacetylation of PNAG

**Fig 3:**
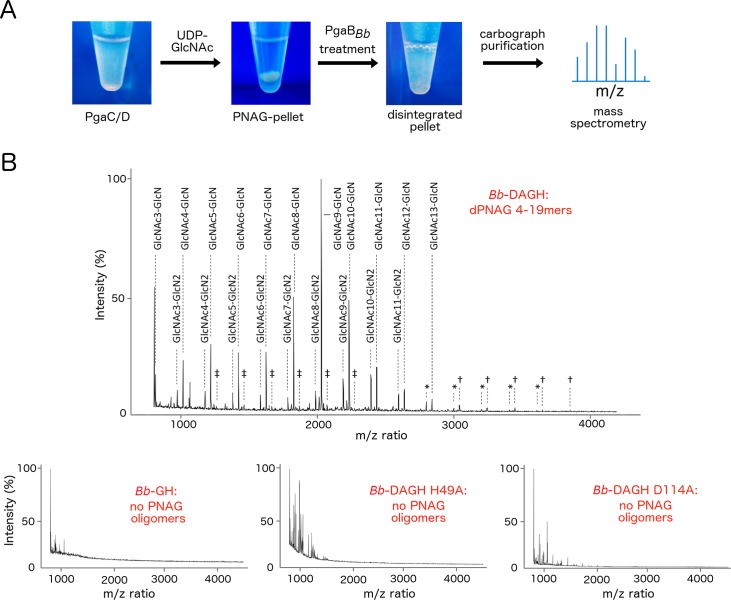
*Bb*-DAGH degrades *in situ* produced PNAG. (A) Schematic of the *in situ* PNAG digestion assay. PNAG is produced *in situ* using PgaCD containing *E*. *coli* membranes. PNAG production and enzymatic cleavage is verified by mass-spectrometry after purification of the hydrolyzed sample. (B) MALDI-TOF MS profiles of product released by PgaB_*Bb*_ variants. The profile of the *Bb*-DAGH treated sample is enlarged to show details in the detected dPNAG/PNAG cleavage products. Profiles of samples containing no detectable dPNAG/PNAG components are displayed in the bottom row. Symbols represent identified structures less than 1% of relative intensity, ‡: fully N-acetylated structures, †: mono-deacetylated structures, *: di-deacetylated structures.

As the isolated and biofilm forms of PNAG are both partially deacetylated, we next set out to determine whether deacetylation of PNAG was required for PgaB glycoside hydrolase activity. To test this hypothesis we developed an assay similar to those utilized previously [21], that allowed us to synthesize fully acetylated PNAG *in situ* using PgaCD_*Ec*_-containing membranes and the UDP-GlcNAc activated sugar precursor as a substrate (Fig 3A). After incubating the reaction mixture for 24 h and centrifugation of the product we noticed that it formed an insoluble pellet that was mechanically resistant to pipetting or crushing. Since control reactions without membranes or substrate did not yield a pellet, we hypothesized that the pellet was primarily constructed of PNAG. As high molecular weight PNAG is insoluble, we reasoned that if we could hydrolyze the polymer into short fragments it could then be analyzed using mass spectrometry (MS). We therefore incubated the pellets with either *Bb*-DAGH or *Bb*-GH and found that the insoluble pellet was only disrupted in the presence of the full-length *Bb*-DAGH protein (Fig 3A, Table 1). We verified the *in situ* production of PNAG oligomers by analyzing the purified products of the hydrolysis reaction by MALDI-MS. This analysis revealed masses consistent with PNAG oligomers between 4–19 residues in length that were predominantly mono- or di-deacetylated (Fig 3B). PNAG oligomer masses could only be detected in *Bb*-DAGH-hydrolyzed samples, suggesting that deacetylation of the polymer by the N-terminal domain of PgaB is required for *Bb*-GH activity on the fully acetylated *in situ* produced PNAG.

**Table 1:** Effect of treating *in situ*-produced PNAG with PgaB.

PgaB construct or variant used	Effect of protein treatment on pellet	Detection of PNAG fragments by mass spectrometry
*Bb*-DAGH	Pellet disintegrated	Yes
*Bb*-GH	No effect	No
*Bb*-DAGH H49A	No effect	No
*Bb*-DAGH D114A	No effect	No

To confirm that PNAG hydrolysis requires the deacetylase activity of PgaB and is not the consequence of increased polymer binding affinity due to the presence of the N-terminal domain, we repeated the hydrolysis reactions with the inactive deacetylase H49A and D114A *Bb*-DAGH variants [14]. These mutants were unable to disintegrate the PNAG pellet and did not produce any MS-detectable products (lower panels in Fig 3B, Table 1). Together these experiments strongly suggest that deacetylation of the polymer is required for the hydrolysis of *in situ* produced PNAG.

### Hydrolysis of dPNAG reveals GlcN-GlcNAc-GlcNAc motif at the reducing end of the polymer

**Fig 4:**
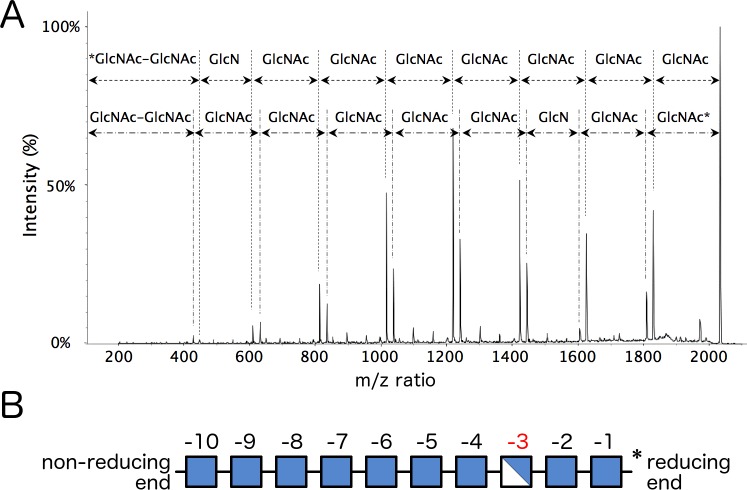
Structural analysis of *Bb*-DAGH treated PNAG reveals a consensus recognition motif. (A) MALDI-TOF MS/MS profile of the m/z ion = 2031.71, a mono-deacetylated PNAG 10-mer. Oligosaccharides were reduced by NaBH_4_ allowing the reducing terminus to be defined. The asterisk indicates the reducing end of the molecule. (B) Graphical representation of the dPNAG 10-mer using the same key as depicted in Fig 1B. Sugar units are numbered relative to the cleavage site at the reducing end.

To determine whether there was a preferred cleavage motif we further characterized the hydrolyzed dPNAG oligomers using MALDI-TOF MS-MS. Fragmentation of the most intense ion at a m/z ratio of 2031.71, representing the reduced mono-deacetylated 10-mer, revealed a (GlcNAc)_7_-GlcN-(GlcNAc)_2_ sequence (Fig 4A). MS-MS fragmentation analysis of all the mono-deacetylated PNAG oligomers observable above the m/z ratio of 1000 revealed that all oligomers had a fragmentation pattern consistent with a GlcN-GlcNAc-GlcNAc motif at their reducing end (Fig 4B and S1 Fig).

The di-deacetylated PNAG oligomers were also analyzed by MS-MS fragmentation (S2A Fig). While structural heterogeneity in these samples precluded unambiguous assignment of the GlcN residues, GlcN was not observed at the reducing end of the oligomers. The spectra are consistent with the two GlcN units positioned between sites -2 and -5 (S2B Fig). While the samples might not contain all possible combinations of sugars at the reducing end, the GlcNAc-GlcNAc-GlcNAc combination can be excluded. The exclusive occurrence of the GlcN-GlcNAc-GlcNAc motif in mono-deacetylated samples suggests that this motif is recognized during dPNAG cleavage. The presence of the same motif in the di-deacetylated oligomers is also likely, although a GlcNAc-GlcN-GlcNAc motif cannot be fully ruled out due to ambiguity in data interpretation.

### The C-terminal domain of PgaB_*Bb*_ defines a new GH family

Based on amino acid sequence, *Bb*-GH and *Ec*-GH (44% sequence identity) have been grouped into the putative GH13-like family [37]. The GH13 family in the CAZy database [38] is a large sequence and structurally diverse family that hydrolyzes substrates containing α-glucoside linkages. Despite their functional diversity, the GH13 family shares a core (β/α)_8_ TIM-barrel fold, and enzymatically active members contain a conserved triad of residues that include a nucleophile (aspartate), an acid/base (glutamate), and a residue that stabilizes the substrate transition state (aspartate). Our previous structural studies revealed that *Ec*-GH had a similar overall structure to GH18, GH20, and GH13 family members, but lacked the respective catalytic consensus motifs found in these GH families [14, 24]. This also holds true for *Bb*-GH, prompting us to initiate structural studies on *Bb*-GH to gain a better understanding of the structure and function of this GH domain.

**Fig 5:**
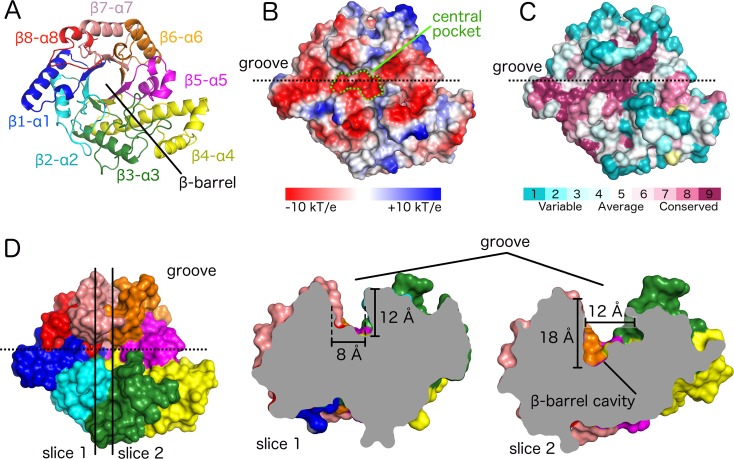
The (β/α)_8_ TIM-barrel of *Bb*-GH has a long and deep groove with a highly conserved central pocket. (A) Cartoon representation of the (β/α)_8_ barrel with each β/α segment shown in a different color. (B) Electrostatic surface representation in the same orientation as panel A. The central pocket forming the deepest region of the groove is highlighted in green. (C) Surface representation with residues coloured based on conservation level (yellow: insufficient data). (D) Surface representation in the same colour coding and orientation as panel A. Slice 1 and 2 display different sections of the groove as indicated by the vertical lines in left panel and viewed from along the groove from left to right.

*Bb*-GH crystallized in the monoclinic space-group *P*2_1_. Diffraction data were collected to 1.76 Å resolution and the structure was determined by molecular replacement. Structural refinement produced a final model with good geometry and *R* factors *R*_work_ and *R*_free_ of 15.1% and 16.7%, respectively (Table 2). Examination of the crystal packing in the asymmetric unit revealed two *Bb*-GH molecules packed in a head to head fashion. We do not believe this assembly is biologically relevant as size exclusion chromatography shows that *Bb*-GH elutes as a monomer in solution (S3A Fig). *Bb*-GH adopts a (β/α)_8_ barrel fold observed in GH13 family members, and also common to a wide range of other glycoside hydrolases families (Fig 5A). Along the top face of the (β/α)_8_ barrel is an extended electronegative groove that is approximately 43 Å long and 11 Å wide. The groove is formed by residues located at the C-terminal end of the core barrel β-strands and the eight β-α-connecting loops (Fig 5A). There is also a defined pocket at the center of the groove (Fig 5B). This central pocket and most of the electrostatic groove contain residues that are highly conserved among orthologues (Fig 5C). The central pocket is the deepest part of the groove; it has a narrow section (slice 1 in Fig 5D) and a wide section (slice 2 in Fig 5D) that contains the center of the β-barrel.

**Table 2:** Summary of data collection and refinement statistics.

	*Bb*-GH
**Data collection**	
Beamline	NSLS X29A
Wavelength (Å)	1.075
Space group	*P*2_1_
Unit-cell parameters (Å,°)	*a* = 72.1, *b* = 91.6, *c* = 75.3, β = 111.5
Resolution (Å)	50.00–1.76 (1.82–1.76)*
Total no. of reflections	740,908
No. of unique reflections	90,334
Redundancy	8.2 (7.1)
Completeness (%)	99.9 (99.3)
Average *I*/*σ*(*I*)	36.6 (7.9)
*R*_merge_ (%)^a^	6.2 (24.9)
**Refinement**	
*R*_work_^b^ / *R*_free_^c^	15.1 / 16.7
No. of atoms	
Protein	5,657
Ligands	57
Water	510
Average B-factors (Å^2^)^d^	
Protein	23.6
Ligands	46.9
Water	36.5
RMS deviations	
Bond lengths (Å)	0.007
Bond angles (°)	1.08
Ramachandran plot^d^	
Total favoured (%)	99.3
Total allowed (%)	100
Coordinate error (Å)^e^	0.16
PDB code	6AU1

^a^*R*_merge_ = ∑∑ | *I* (k)—<*I*>| / ∑ *I* (k) where *I* (k) and <*I*> represent the diffraction intensity values of the individual measurements and the corresponding mean values. The summation is over all unique measurements.^b^*R*_work_ = ∑ ||F_obs_|—k|F_calc_|| / |F_obs_| where F_obs_ and F_calc_ are the observed and calculated structure factors, respectively.^c^*R*_free_ is the sum extended over a subset of reflections (2.2%) excluded from all stages of the refinement. ^d^As calculated using MolProbity [39].^e^Maximum-Likelihood Based Coordinate Error, as determined by PHENIX [40]

*Values in parentheses correspond to the highest resolution shell.

**Fig 6:**
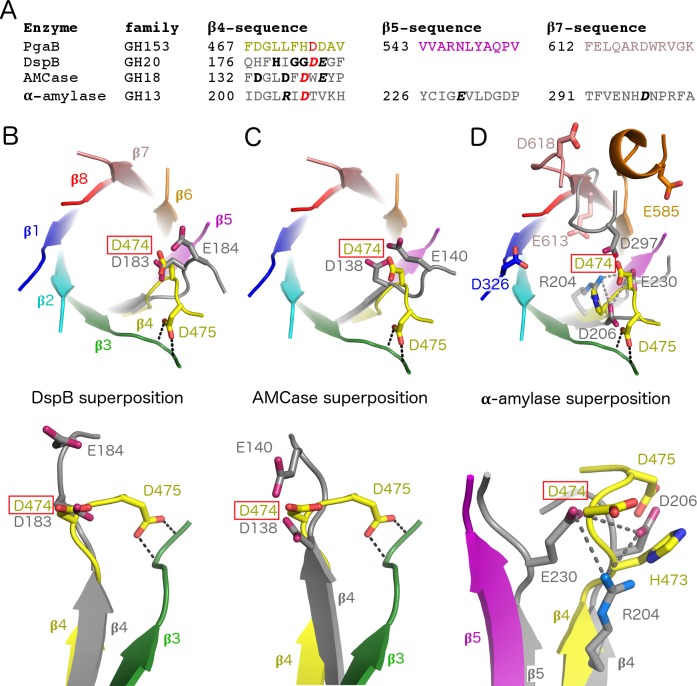
Residue D474 aligns with the catalytic aspartate from structurally similar GH families. (A) Sequence comparison of regions containing catalytic residues. Residues in bold form the consensus motif for each GH family. Residues in bold italics are the catalytic residues. D474 and the structurally aligned aspartates are highlighted in red. Superposition of *Bb*-GH with (B) DspB (PDB 1YHT) [42], (C) AMCase (PDB 2YBU) [81], and (D) α-amylase (PDB 7TAA) [82]. *Bb*-GH β-strands are shown in the same color scheme as Fig 5A, all other enzymes are shown in grey.

Surveying the PDB for structurally related proteins using the DALI webserver [41] returned results similar to those previously reported for *Ec*-GH [23, 24]. The CAZy database groups glycoside hydrolases based on their primary sequence [38], however members of a given GH family act on similar (if not the same) substrate(s). The top hits had good Z-scores of 19–24 and were predominately from families GH42, GH35, GH14, GH18, and GH20. The GH20 family contains the only currently characterized PNAG hydrolase, DspB [31–33, 42, 43]. Superposition of *Bb*-GH and DspB revealed that D474 of *Bb*-GH aligns with the catalytic aspartate residue of DspB, D183. However, *Bb*-GH lacks a structural equivalent to the second catalytic residue of DspB, E184 (Fig 6A and 6B). A similar scenario is observed when *Bb*-GH is superposed with the GH18 chitinase AMCase (Fig 6C). *Bb*-GH also displays significant structural differences when compared to the canonical representative of the GH13 family, α-amylase. *Bb*-GH does not contain the D206, E230, and D297 catalytic triad residues found in α-amylase (Fig 6D). Most notable is the absence of a residue that contributes to the stabilization of the transition state. In *Bb*-GH the closest aspartate and glutamate residues D618, E613, E585, and D326 are over 8 Å displaced and thus unlikely to replace D297. Considering that neither PgaB_*Bb*_ nor PgaB_*Ec*_ have sequence or structural similarity to the active site to DspB or other closely related GH family members, we propose that the C-terminal domain of PgaB defines a new GH family, GH153.

### Residues in the elongated binding groove are critical for function

**Fig 7:**
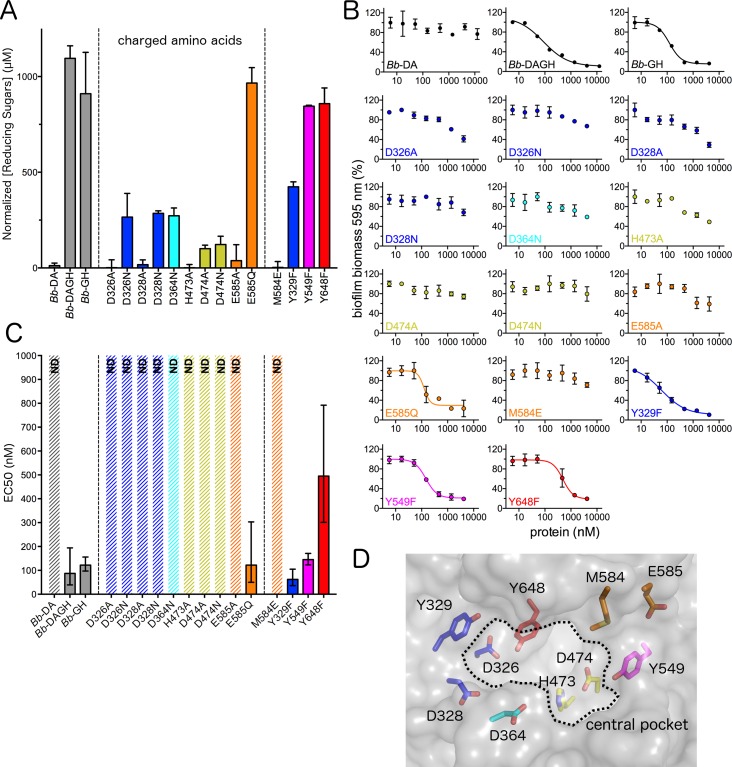
Mutagenesis suggests D474 is involved in catalysis and other charged residues crucial for dPNAG binding. (A) Reducing sugar assay with 2 mg/ml dPNAG purified from *S*. *aureus* with 2 μM PgaB constructs over 24 h. Error bars, S.E. from two independent experiments performed in duplicate. (B) *E*. *coli* biofilm disruption assay. Error bars represent the S.E. with n = 3. (C) EC_50_ values from *E*. *coli* biofilm disruption assay. ND: EC_50_ not determined as > 50% biomass remained or no plateau was reached after treatment with 5 μM enzyme for 2 h. Error bars show 95% confidence interval, and the same color scheme in A is used. (D) Transparent surface representation of the *Bb*-GH binding groove with mutated amino acids shown in stick representation. Note that D364 is fully buried and only visible due to the transparent representation.

Given our hypothesis that substrate recognition and the PgaB_*Bb*_ active site differ from previously characterized glycoside hydrolases, we utilized mutagenesis to determine which residues are important for dPNAG hydrolase activity. Initially, we constructed a set of *Bb*-GH mutants focusing on conserved charged residues in the active site groove within 10 Å of the central cavity. The function of each mutant was evaluated using the reducing sugar and biofilm disruption assays. The reducing sugar assay revealed that mutation of D326, D328, H473, D474, and E585 to alanine reduced the activity of the enzyme by more than 90% (Fig 7A). We were unable to assess the activity of the D364A variant as this protein was unstable and aggregated during purification. To help differentiate between residues involved in substrate binding and catalysis, we also mutated the charged residues to either asparagine or glutamine as appropriate. An increase in activity relative to the alanine variant was observed for D326N, D328N and E585Q, with E585Q recovering wild-type levels of activity. No increase in activity was observed for the D474N mutant relative to its alanine counter-part. The D364N mutant exhibited ~25% of wild-type activity comparable to the activity observed for the D326N and D328N mutants.

The central cavity of the electronegative groove also contains three tyrosine residues and a methionine. To probe the role of these residues, tyrosine to phenylalanine and methionine to glutamate variants were constructed. Mutation of Y549 and Y648 to phenylalanine had little effect on the activity, however, the Y329F variant showed a 2-fold reduction in activity relative to wild-type. Although we had hypothesized that replacing M584 with glutamate may aid in the binding or catalysis of dPNAG, the M584E variant was completely inactive (Fig 7A). We further analyzed the *Bb*-GH variants using our biofilm disruption assay, to determine whether differences in the activity would be observed when *E*. *coli* dPNAG was used as the substrate. We observed that results from the biofilm disruption assay correlate well with the reducing sugar assay. In general, variants with less than 30% residual activity in our reducing sugar assay were unable to disrupt biofilms (Fig 7B and 7C). Taken together, the mutagenesis data suggests that residues D326, D328, D364, and E585 play a role in substrate binding as the asparagine or glutamine mutants displayed detectable activity, while D474 is the most probable residue to be involved in catalysis as it is the only residue whose activity is ablated in both the asparagine and alanine variants.

### *Bb*-GH has a more open active site pocket compared to *Ec*-GH

**Fig 8:**
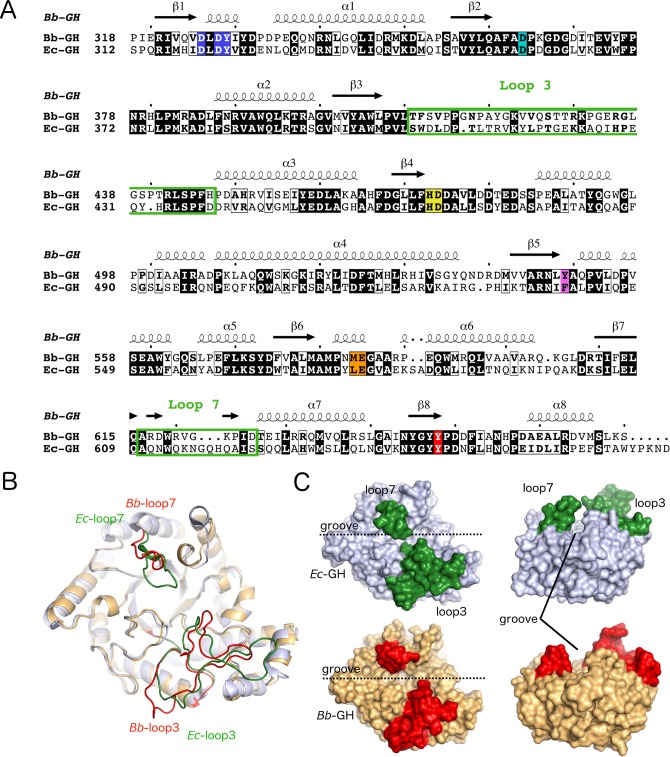
Sequence and structural comparison of *Ec*-GH and *Bb*-GH reveal differences in active site accessibility. (A) Sequence alignment between *Bb*-GH and *Ec*-GH showing identical and similar residues shaded and boxed in black, respectively. Secondary structure elements of *Bb*-GH are shown above the sequence alignment with the canonical (β/α)_8_ elements labeled. Residues forming the active site pocket and analyzed by mutagenesis are highlighted with the same color scheme as in Fig 7, with loops 3 and 7 highlighted by green boxes. The sequence alignment figure was generated using ESPript 3.0 [83]. (B) Structural superposition of *Bb*-GH and *Ec*-GH shown in cartoon representation. (C) Surface representation of *Ec*-GH and *Bb*-GH. In (B) and (C) loops 3 and 7 are highlighted in red and green, respectively.

Our hydrolase activity analysis revealed that *Bb*-GH is 4 times more active than *Ec*-GH (Fig 1C), however, a pairwise sequence alignment does not show any major difference in the residues tested by our mutagenesis analysis that could explain this result (Figs 7D and 8A). Structural alignment of *Bb*-GH (PDB 6AU1) and *Ec*-GH (PDB 4P7L) shows strong conservation with a root mean square deviation of 1.1 Å over 337 equivalent Cα atoms (Fig 8B). The most significant structural differences are seen in loop 3 and 7, which are involved in forming the sidewalls of the central pocket (Fig 8B and 8C). Loop 7 in *E*. *coli* PgaB extends much further over the groove than in *B*. *bronchiseptica*, almost completely occluding the binding groove (Fig 8C). Loop 3 is the longest loop in the orthologous PgaB structures and folds back into the active site pocket (Fig 8C). Examination of the residues in loop 3 reveal significant amino-acid variation between *Bb*-GH and *Ec*-GH that may partially explain the difference in activity between the two enzymes. Although it is hard to predict the conformational flexibility of these loops, these findings suggest that a more restricted active site groove in PgaB_*Ec*_ might lead to reduce hydrolase activity due to lower binding affinity or reduced accessibility for dPNAG.

### Individual sugar binding sites support dPNAG recognition in the elongated binding groove

**Fig 9:**
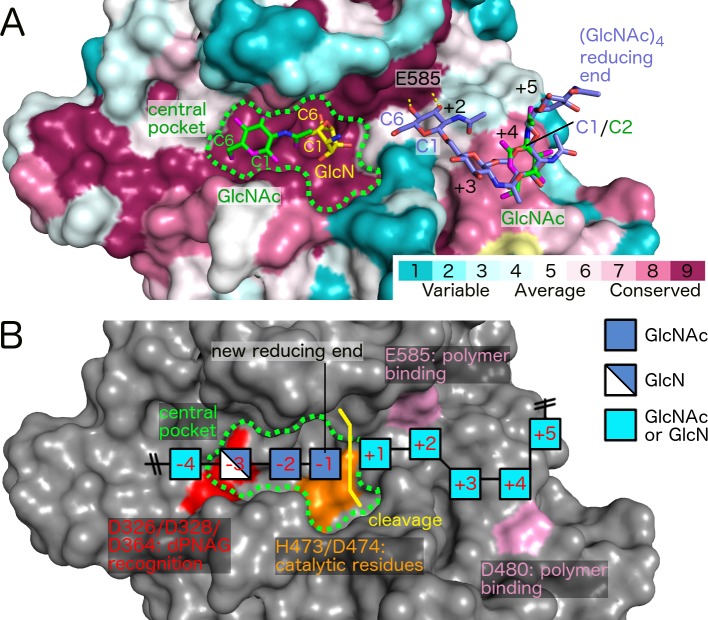
Proposed recognition of dPNAG required for polymer cleavage by *Bb*-GH. (A) Surface representation of *Bb*-GH with residues coloured based on conservation level (yellow: insufficient data). Shown in stick representation are GlcN (PDB 4P7N), GlcNAc (PDB ID 4P7Q), and (GlcNAc)_4_ (PDB ID 4P7R) that have been co-crystallized with *Ec*-GH and were modeled into the *Bb*-GH structure. (B) Proposed cleavage mechanism based on mutagenesis and mass spectrometry analysis. Crucial surfaces are highlighted in red, orange, and pink. Positions -4 to +5 denote expected binding sites of GlcN/GlcNAc units relative to the cleavage site. Positions +2 to +5 were taken directly from (GlcNAc) _4_, position -1 coincides approximately with a GlcN monomer bound to PgaB_*Ec*_ (PDB 4P7N) and position -3 with a GlcNAc monomer bound to PgaB_*Ec*_ (PDB 4P7Q) [24].

Structures of *Ec*-GH bound to GlcNAc and GlcN monomers and a GlcNAc tetramer (GlcNAc)_4_ have been determined previously [24]. Since the groove region is highly conserved across PgaB enzymes, we modeled these ligands into the *Bb*-GH structure to gain insight into the catalytic mechanism and how the GlcN-GlcNAc-GlcNAc motif is recognized by the enzyme (Fig 9A). The (GlcNAc)_4_ binding site is formed mainly by the loop region between β5 and α5 (Fig 5A). The oligosaccharide is oriented with its non-reducing end closest to the central pocket (Fig 9A). The O3 and O4 hydroxyls of the GlcNAc unit closest to the central pocket form hydrogen bonds with carboxylate side chain of E585 (Fig 9A). This interaction is compatible with our mutagenesis data that suggests E585 plays a role in polymer binding but not catalysis. Given the location of the catalytic reside D474 and the distance to E585, we hypothesize that the (GlcNAc)_4_ oligomer occupies positions +2 to +5 relative to the site of cleavage (-1/+1) (Fig 9B). Reminiscent of position +2, the oligomeric GlcNAc unit at position +4 forms hydrogen bonds via O3 and O4 hydroxyls to the carboxylate side chain of D480, suggesting that this residue plays a role in polymer binding similar to E585 (Fig 9B).

Modeling of the GlcN and GlcNAc monomers identifies the -3 and -1 sugar binding sites. GlcN and GlcNAc monomers bind in the central pocket interacting with the proposed catalytic residue D474, and three aspartate residues, D326, D328 and D364, respectively (Fig 9A, S4 Fig). An additional GlcNAc monomer also binds to the +4 site (Fig 9A). Examination of this GlcNAc reveals that its orientation is not the same as observed in the (GlcNAc)_4_ oligomer, and not compatible with polymer binding. This is perhaps not unexpected as monomeric sugar units when binding to the protein would be able to adopt the most favourable conformation possible without strict constraints imposed when part of a longer polymer. Inspection of the individual sugar moieties in the -3 and -1 positions also suggests that while the sugar identifies a binding site, the orientations are unlikely compatible with polymer binding. For example, the C6 atom of GlcN that participates in the 1,6-linkage in the PNAG polymer is pointing towards the β-barrel cavity and not along the groove. In addition, the C1 atom, which would define the reducing end of a polymer, would result in a polymer that is oriented in the opposite direction relative to (GlcNAc)_4_. In the case of GlcNAc, the position of atom C1 would result in a polymer that has the same orientation as the (GlcNAc)_4_ oligomer, but the acetyl-group is oriented along the groove and would clash with the GlcN in the -3 site (S4 Fig). As demonstrated by the GlcNAc unit at the +4 site, we assume that monomeric sugar compounds indicate approximate binding sites of polymeric sugar units, but not necessarily the correct orientation. Combining the individual ligand binding sites suggest that a dPNAG oligomer containing 9 sugar units binds along the elongated and conserved binding groove of PgaB (Fig 9B).

## Discussion

Treating chronic biofilm-associated infections is a significant medical problem. A better understanding of mechanisms involved in biofilm formation as well as new methods to disrupt or inhibit biofilms that render the bacteria more susceptible to antimicrobial agents and host defense mechanisms are in urgent need. In this report, we show that PgaB can hydrolyze dPNAG and demonstrate its potential use as a biofilm disruption agent.

Our previous characterization of PgaB led to the hypothesis that the C-terminal domain was catalytically inactive [23, 24]. However, leveraging our experience from investigating other exopolysaccharide systems, we show herein that the C-terminal domain of PgaB is a glycoside hydrolase that can cleave dPNAG. The presence of an active GH or carbohydrate-cleaving enzyme within bacterial EPS biosynthetic operons or fungal EPS gene clusters is an emerging trend [20, 44]. The biological role of EPS-cleaving enzymes within the biosynthesis machinery is not fully understood and appears to be system specific. In some cases, such as for *Acetobacter xylinum* BcsZ [26], *A*. *fumigatus* Sph3 [29], and *P*. *aeruginosa* AlgL [28], the enzyme is required for or imparts efficient exopolysaccharide biosynthesis and/or export. While in other cases the enzyme is dispensable for biofilm formation, like the *P*. *aeruginosa* PSL-polysaccharide hydrolase PslG [30]. However, overexpressing PslG results in impaired biofilm formation and less surface associated Psl, suggesting intracellular levels are critical during the Psl biosynthetic process [30]. Various functions for these carbohydrate-cleaving enzymes have been proposed, such as controlling polymer length, generating a secretion-competent form of the polymer, as well as degradation of excess polymer in the periplasm [25, 45, 46]. Previous studies showed complementation of a Δ*pgaB* strain with constructs lacking part of, or all of the C-terminal domain resulted in cell-retained material and abolished biofilm formation [46]. This suggests that the glycoside hydrolase activity of PgaB may be required for biofilm formation in *E*. *coli*. However, this interpretation is complicated by the observation of proteolytic degradation of the truncated PgaB constructs [46] and the fact that deacetylase activity of the N-terminal domain of PgaB_*Ec*_ requires the C-terminal GH domain [24]. As the PgaB_*Ec*_ N-terminal domain is not active in isolation, the observed phenotype could have arisen from the loss of deacetylase activity. This conclusion is supported by alanine mutagenesis studies on the C-terminal domain of the *Y*. *pestis* PgaB homologue, PgaB_*Yp*_ (also known in the literature as HmsF) as mutating conserved residues, equivalent to those required for dPNAG hydrolysis herein (D364 and D474), showed a normal biofilm phenotype [47]. However, phenotypic defects from single alanine variants can easily be masked by the increased gene copy number from complementing *in trans*. Interestingly, in *Salmonella enterica* the cellulase BcsZ, part of the cellulose biosynthesis system, has been reported to efficiently enable host colonization by functioning as a negative regulator of cellulose biosynthesis [48]. Negative regulation of biofilm related cellulose production has been linked earlier to increased virulence in *Salmonella* species [49], indicating a role in balancing short- and long-term fitness. Further studies will be necessary to uncover the exact role for PgaB-dependent hydrolysis of dPNAG during biosynthesis, export, and biofilm maturation.

In addition to functioning as a GH, exopolysaccharide-cleaving enzymes that reside in the periplasm have also been suggested to form part of larger protein complexes within the biosynthetic machinery. This provides a scaffold for the polymer to cross the periplasmic space bridging the inner and outer membrane components [20, 44, 50]. In *E*. *coli*, PgaB forms a complex with the periplasmic TPR domain of the outer membrane porin, PgaA [22]. This interaction appears to be critical as deletion of the TPR domain abolishes biofilm formation [22]. A similar interaction between PgaA_*Yp*_ and PgaB_*Yp*_ (also known as HmsH and HmsF), the *Y*. *pestis* homologues, has also been observed using *in vivo* cross-linking studies [51]. Similarly, a recent study from our lab on the PEL polysaccharide system showed that PelA, an enzyme with PEL deacetylase and glycoside hydrolase activities, interacts with the TPR domain of the outer membrane porin PelB [52]. PelB has the same domain architecture as PgaA. Furthermore, when the activities of PelA were assessed *in vitro*, we found that the interaction of the TPR domain of PelB with full length PelA resulted in increased deacetylase and decreased glycoside hydrolase activity compared to PelA alone [52]. Considering these findings, it is tempting to speculate that the TPR domain of PgaA could also modulate the enzymatic activities of PgaB and regulate the temporal modification of PNAG during biosynthesis.

Our previous molecular dynamic simulations [24] suggested a continuous inter-domain PNAG binding surface, and that the C-terminal GH domain would preferentially bind dPNAG over PNAG. Therefore, we postulated that the PNAG polymer is first deacetylated and then wraps around PgaB to the GH domain prior to export through PgaA. Unfortunately we were not able to determine the exact level of deacetylation in small-scale biofilms due to the low solubility of PNAG, which makes it difficult to unambiguously prove that deacetylation is required for hydrolysis in these assays. Instead, we used the PgaCD synthase complex to produce fully acetylated PNAG *in vitro*, and subsequently tested the effect of PgaB deacetylation on glycoside hydrolase activity. Our mass spectrometry data strongly suggests that the deacetylation of PNAG is required for PgaB glycoside hydrolase activity. This conclusion is supported by our hydrolase assays using *in situ* produced PNAG (i.e. fully acetylated), which was only cleaved by full-length *Bb*-DAGH and not the isolated *Bb*-GH domain (Fig 3). However, until longer soluble PNAG oligomers are available, including those with single site-specific deacetylation of the polymer, we cannot completely rule out the possibility that PgaB could display glycoside hydrolase activity on fully acetylated PNAG. Our data also suggest a model where *Bb*-GH is an *endo*-acting GH, and is supported by MS/MS data that detected dPNAG oligomer cleavage products of 4 to 19 sugar units in length from the hydrolase assays. Furthermore, *Bb*-GH and *Ec*-GH lack hydrolytic activity on *p*NP-glycoside substrates and short PNAG oligomers and contain a pronounced and well-conserved substrate-binding groove suited to binding long stretches of polymer [24]. This is in contrast to the only other characterized PNAG hydrolase, DspB, which displays both *exo* and *endo*-acting hydrolysis of PNAG and has a shallow substrate-binding groove [53, 54]. However, it does correlate with the bacterial cellulose and Psl biosynthesis systems, as BcsZ and PslG are both *endo*-acting glycoside hydrolases with long and deep substrate-binding clefts [25, 26].

MS/MS analysis of the hydrolyzed dPNAG oligomers revealed unambiguously a GlcN-GlcNAc-GlcNAc motif at the reducing end of mono-deacetylated cleavage products, suggesting that the polymer orients itself in the binding groove with GlcN and GlcNAc sugar units positioned at sites -3 and -1 during catalysis, respectively (Fig 9B). The position of the positively charged GlcN could be stabilized at site -3 by the negatively charged aspartate residues D326/D328/D364. This is supported by our mutagenesis study that show residues in the -3 site are important for activity and suggests they may be involved in pre-orientating the polymer for cleavage at site -1 by the proposed catalytic residue D474 (Figs 7 and 9B). Furthermore, the -3 site is located at the narrowest part of the groove (Fig 5D) and steric hindrance could therefore play a role in substrate specificity as the *N*-acetyl group may clash with residue side chains that line the wall.

The observed hydrolysis of dPNAG by the GH domain of PgaB (Fig 1C) is consistent with the observation that the secreted form of PNAG found in bacterial biofilms is partially deacetylated [34, 55]. The extent of deacetylation depends on the bacterial species and growth conditions, but has been reported to be between 3–25% [34, 46, 55]. Furthermore, we exploited the dPNAG hydrolase activity of *Bb*-GH to disrupt biofilms formed by PNAG-overproducing strains of *B*. *pertussis*, *S*. *carnosus*, and *E*. *coli* as well as a clinical isolate of *S*. *epidermidis* with EC_50_ values in the nanomolar range (Fig 1D and 1E). This is underscored by the ability of PgaB to disrupt biofilms in the presence of serum and potentiate bacterial killing by gentamicin (Fig 2). The ability to disrupt biofilms formed by a variety of different Gram-negative and Gram-positive bacteria suggests that, like the previously characterized glycoside hydrolases DspB [31–33], PelA [56, 57], PslG [30, 56], Sph3 [29, 57], NghA [58], and PssZ [27], PgaB may have therapeutic potential for treatment of a broad range of infections caused by PNAG-producing bacteria such as *E*. *coli* [34], *Staphylococcus spp*. [55, 59, 60], *Bordetella* spp. [6, 7], and others [19, 33, 61–66]. In direct comparison PgaB displays a higher EC_50_ value than DspB (Fig 2A), which might be related to their individual biological roles or their substrate specificity. While we have no evidence to propose that PgaB plays a biological role in biofilm dispersal, a surface self-dispersal mechanism is employed by *Aggregatibacter actinomycetemcomitans* and potentially by *Yersinia pseudotuberculosis* that express DspB and NghA, respectively [58].

*Bb*-GH illustrates the challenge of categorizing glycoside hydrolases based solely on primary sequence without structural or functional characterization, as our data strongly suggest that *Bb*-GH comprises a new GH family, GH153. Furthermore, the structural and functional characterization of PgaB presented herein suggests that the mechanism of periplasmic processing and outer-membrane export of dPNAG polymer involves deacetylation and limited hydrolysis in concurrent order, and establishes *Bb*-GH as a potential therapeutic agent for selective treatment of PNAG-dependent biofilm infections.

## Materials and methods

### Cloning, expression, and purification of PgaB constructs and DspB

Bacterial strains, plasmids, and oligonucleotide primers used in this study are described in Table A in S1 Text. PgaB_*Bb*_ constructs were derived from *B*. *bronchiseptica* RB50 (accession number WP_010926292), and the C47S variant of PgaB_*Bb*_ was used for the *Bb*-DAGH and *Bb*-DA constructs to eliminate non-specific cysteine cross-linking during purification. The C-terminal domain from PgaB_*Bb*_ encoding residues 318–670 was cloned into the pET28a expression vector using PCR with pET28-*Bb*-DAGH as template and primers 318 Fwd and 670 Rev that contain an NdeI and HindIII site, respectively. The resulting plasmid pET28-*Bb*-GH, encodes a thrombin-cleavable N-terminal hexa-histidine tag fused to *Bb*-GH. The *Bb*-GH variants were all cloned using the QuikChange Lightning site-direct mutagenesis kit using plasmid pET28-*Bb*-GH as template and the respective variant primers listed in Table A in S1 Text. All proteins were expressed and purified as described previously for *Bb*-DAGH [14] and *Ec*-DAGH [67]. The purified PgaB_*Bb*_ and PgaB_*Ec*_ constructs were >95% pure as judged by SDS-PAGE and stable for approximately 2 weeks at 4°C or 6 months at -20°C.

A codon-optimized gene encoding DspB (accession number AAP31025) was purchased from BioBasic and sub-cloned into the pET24a expression vector using the NdeI/XhoI restriction sites. The resulting plasmid pET24a-DspB encodes a non-cleavable C-terminal hexa-histidine tag fused to DspB, which was used to express and purify DspB as described previously [31].

### *p*Nitrophenyl-glycoside hydrolase assays

To probe for GH activity, the following artificial glycoside hydrolase substrates were tested: *p*NP-α-galactose, *p*NP-β-galactose, *p*NP-α-GalNAc, *p*NP-β-GalNAc, *p*NP-α-glucose, *p*NP-β-glucose, *p*NP-β-mannose, and *p*NP-β-GlcNAc. The *p*NP glycoside substrates were dissolved in DMSO at a concentration of 50 mM. The enzymatic reaction (100 μl) contained 2.5 mM *p*NP glycoside substrate and 40 μM *Bb*-GH in 100 mM HEPES buffer pH 7.0. Reactions were initiated by the addition of substrate and allowed to proceed for 120 min. The reaction was monitored for the appearance of 4-nitrophenyl in real time at 405 nm.

### Purification and re-suspension of dPNAG

The extraction, purification, and level of deacetylation of dPNAG from *S*. *aureus* strain MN8m were performed as described previously [62, 68]. Lyophilized dPNAG was dissolved in ice-cold 5 N HCl by pipetting and vortexing. The solution was then placed on ice and titrated to neutrality (pH 7–8) by slowly adding ice cold 5 N NaOH. The final solution of dPNAG was slightly turbid and ranged between 3–6 mg/ml.

### dPNAG reducing sugar assay

Determination of carbohydrate reducing-ends was performed as described previously [69]. Briefly, in a 50 μl reaction 2 μM of a PgaB construct or DspB was incubated with 2 mg/ml of dPNAG in 100 mM HEPES pH 7.0 for 2 or 24 h. The sample was split into two 20 μl aliquots and treated with 0.5 M NaOH, then the dithiotreitol/3-methyl-2-benzothiazolinone hydrozone solution (1:3 mg/ml ratio), and heated at 80°C for 15 min. A solution containing 0.5% ammonium iron(III) sulfate, 0.5% sulfamic acid, and 0.25 N HCl was added, mixed, and cooled to room temperature. A volume of 100 μL was then transferred to a 96-well clear bottom plate and the absorbance was measured at 620 nm using a SpectraMax M2 plate reader (Molecular Devices, Sunnyvale, CA). Protein and dPNAG in 100 mM HEPES pH 7.0 were used as background controls. GlcN solutions were used to determine a standard curve to determine the concentration of reducing sugars.

### Biofilm disruption assays

*E*. *coli* DH5α biofilm assay: *E*. *coli* DH5α containing plasmid pMM11 was grown overnight in LB broth supplemented with 40 μg/ml chloramphenicol with shaking at 200 rpm, diluted to an OD_600_ of 0.05 into fresh media, and 100 μl was added to individual wells of 96 well cell culture plates (Costar 3596) and incubated statically for 24 h at 25°C. Non-adherent cells and media were removed by washing the plate three times with deionized water. The wells were then incubated with 100 μl of 100 mM HEPES pH 7.0 containing 1.3 μM of PgaB_*Bb*_ at room temperature for 2 h with gentle shaking. The wells were then washed three times with deionized water, stained with 150 μl of 0.1% (w/v) crystal violet for 10 min, and washed again three times with deionized water. The remaining dye was solubilized with 100 μl of 95% (v/v) ethanol for 10 min with rotation, after which time the absorbance was measured at 540 nm.

*B*. *pertussis* 536 biofilm assay: *B*. *pertussis* strain 536 Δ*bps* carrying plasmid pMM11 was grown overnight in Stainer Scholte (SS) media supplemented with 10 μg/ml chloramphenicol, diluted to an OD_600_ of 0.1 into fresh media, and 250 μl of normalized culture was added to individual chambers of coverglass slides (8 chamber Lab Tek II coverglass system) and incubated for 72 h at 37°C. Non-adherent cells and media were removed by washing the chamber three times with deionized water. Chambers were then incubated with 250 μl of 100 mM HEPES pH 7.0 containing 1.3 μM of PgaB_*Bb*_ at room temperature for 2 h. The reaction was then quenched by washing the coverslides three times with deionized water, and the remaining biomass was quantified using crystal violet as described above for *E*. *coli* DH5α biofilm.

*S*. *epidermidis* biofilm assay: Cultures of *S*. *epidermidis* clinical isolate SE801 were grown overnight at 37°C with shaking at 200 rpm in LB broth without antibiotics. The next day cultures were sub-cultured 1:100 into tryptic soy broth (TSB), mixed thoroughly and 100 μl was added to individual wells of a sterile Cellbind surface plate (Corning) and the plates were incubated statically for 24 h at 26°C to allow for biofilm formation. To eliminate edge effects, ~200 μl of sterile water was placed in all outside wells and the plate was sealed with parafilm. After incubation non-adherent cells and media were removed by washing the plate with deionized water three times. The wells were filled with 95 μl of 100 mM HEPES buffer pH 7.0 followed by 5 μl of varying concentrations of protein. Reactions were incubated for 2 h at 25°C on a rotating nutator at which time, the reaction was quenched by washing the plates with deionized water three times. The wells were then stained with crystal violet, solubilized, and quantified as outlined above for DH5α biofilms but using 595 nm as the wavelength.

*S*. *carnosus* TM300 biofilm assay: Cultures of *S*. *carnosus* TM300 containing pTX*ica*ADBC were grown overnight at 37°C with shaking at 200 rpm in LB broth supplement with 5 μg/ml tetracycline. The next day cultures were sub-cultured 1:100 into tryptic soy broth (TSB) supplemented with 0.5% (w/v) xylose and 5 μg/ml tetracycline, mixed thoroughly and 100 μl was added to individual wells of a sterile Cellbind surface plate (Corning) and the plates were incubated statically for 24 h at 26°C to allow for biofilm formation. To eliminate edge effects, ~200 μl of sterile water was placed in all outside wells and the plate was sealed with parafilm. After incubation non-adherent cells and media were removed by washing the plate with deionized water three times. The wells were filled with 95 μl of 100 mM HEPES buffer pH 7.0 followed by 5 μl of varying concentrations of protein. Reactions were incubated for 2 h at 25°C on a rotating nutator at which time, the reaction was quenched by washing the plates with deionized water three times. The wells were then stained with dye, solubilized, and quantified as outlined above for DH5α biofilms but using 595 nm as the wavelength.

*E*. *coli* K-12 biofilm assay: Cultures of *E*. *coli* K-12 TRXWMGΔABCD complimented with pPGA372 were grown overnight at 37°C with shaking at 200 rpm in LB broth supplemented with 100 μg/ml kanamycin and 200 μg/ml ampicillin. Overnight cultures were sub-cultured 1:100 into LB broth supplemented with antibiotics, mixed thoroughly, and 100 μl was added to individual wells of a sterile 96-well polystyrene round bottom microtiter plate (BD Falcon). The cultures were incubated statically for 24 h at 26°C to allow for biofilm formation. Biofilm formation quantification and dose response curves were determined as described above for the DH5α (540 nm) and *S*. *epidermidis* (595 nm) biofilms, respectively.

Biofilm disruption assays including serum were conducted in 96-well Cellbind surface plates (Corning) as described above for *S*. *epidermidis* SE801 and *E*. *coli* K-12 strains but with 10% fetal bovine serum (FBS) added in the disruption solution containing TSB and varying concentrations of *Bb*-GH or DspB.

### Antibiotic potentiation assays

Biofilms were grown for over 24 h in 96-well Cellbind surface plates (Corning) as described previously for the *S*. *epidermidis* SE801 and *E*. *coli* K-12 biofilm disruption assays but using TSB as growth media for both strains. TSB was removed and biofilms were washed twice with PBS. Reactions were performed in TSB supplemented with 10 μM *Bb*-GH or DspB and 500 or 50 μg/ml gentamicin for *S*. *epidermidis* SE801 and *E*. *coli* K-12 biofilms, respectively. Cultures that were not treated or were only treated with hydrolase or antibiotic served as controls. Reactions were incubated for 20 h (*S*. *epidermidis* SE801) and 4 h (*E*. *coli* K-12) at 37 **°**C. Afterwards, for reactions in the presence of hydrolase the reaction media was serially diluted in PBS. For reactions in the absence of hydrolase the supernatant was removed and kept on ice while biofilms exposed to 10 μM *Bb*-GH or DspB to disrupt the biofilm-embedded bacteria. After 10 min the suspended cells and supernatant were combined and serially diluted in PBS. Dilutions were plated on LB agar plates at 37 **°**C and enumerated after 24 h.

### Production and hydrolysis of in situ produced PNAG

*E*. *coli* K-12 *pgaC* and *pgaD* were cloned into the pETDuet-1 co-expression vector using PCR with pPGA372 as template, restriction digest, and ligation cloning (BamHI and HindIII, and NdeI and XhoI sites, respectively). This produced the expression plasmid pETDuet-PgaCD that encodes for the co-expression of PgaC with a non-cleavable N-terminal His-6 tag and PgaD. The *wspR*-R242A variant was subcloned from pET28-WspR R242A [70] into the pET24 vector between the NdeI and XhoI sites. This produced the expression plasmid pET24-WspR R242A that encodes WspR R242A without a purification tag. BL21 CodonPlus cells transformed with pETDuet-PgaCD and pET24-WspR R242A were grown overnight at 37°C with shaking in LB with 100 μg/ml ampicillin and 50 μg/ml kanamycin, and then sub-cultured into 1 L LB and grown to an OD_600_ = 0.4–0.5. The culture was moved to 18°C for 20–30 min until the OD_600_ = 0.6–0.7 and was induced with 1 mM IPTG and incubated overnight. Cells were harvested by centrifugation at 5,000 x *g* for 15 min, re-suspended in 40 ml of PgaCD lysis buffer (50 mM HEPES pH 7.5, 300 mM NaCl, 1 mM TCEP, 5% (v/v) glycerol, and a protease tablet), and lysed by homogenization through an Emulsiflex-c3 (4 passes, 10-15K psi). Cellular debris was removed by centrifugation at 20,000 x *g* for 30 min, and the supernatant was further centrifuged at 200,000 x *g* for 60 min to pellet the membranes. Membranes were isolated and homogenized in a hand press twice in PgaCD lysis buffer with 20% (v/v) glycerol. Aliquots (50–100 μL) were placed into 1.5 mL tubes and utilized immediately in assays or stored at -20°C until required. Production of c-di-GMP for the hydrolase assays was synthesized and purified as described previously [70]. PgaCD membrane aliquots were incubated with 10 mM UDP-GlcNAc, 5 mM MgCl_2_, and 1 mM c-di-GMP to a final volume of 200 μl. Tubes were allowed to sit for 24 h at 30°C and after 5 min of centrifugation at 7,400 x g on a table-top centrifuge a pellet was formed. Pipetting lifted the pellet from the Eppendorf tube bottom and it was washed three times with water. The pellet was then incubated to 200 μl of 100 mM HEPES buffer pH 7.0 and 2 μM PgaB protein for 12 h. Reactions were quenched by purification as described below.

### Purification and mass spectrometry of enzymatically hydrolyzed dPNAG/PNAG

Samples were purified by modifying a protocol used for the purification of alginate oligosaccharides [71]. Reactions were quenched by applying the entire reaction to a pre-equilibrated Grace Alltech Extract-Clean Carbograph column. The column was washed with 6 ml of dH_2_0, 25% (v/v) acetonitrile, and 100% (v/v) acetonitrile. All fractions were then dried using a speed vacuum and reconstituted in 0.2% (v/v) trifluoroacetic acid (TFA) for MALDI-TOF MS. MS/MS analysis was ran after reduction of the sample. Samples were incubated overnight in 1 mg/ml NaBH_4_ in 1 M NH_4_OH prior to being neutralized with acetic acid and purified on Hypersep Hypercarb SPE column (ThermoFisher) and eluted with 50% (v/v) acetonitrile. Samples were spotted in a 1:1 ratio with 5 mg/mL of 2,5-dihydroxybenzoic acid matrix in acetonitrile-0.2% TFA (70:30, v:v). Analysis was performed on the UltrafleXtreme MALDI-TOF/TOF in positive reflector mode recording 5,000 laser shots per sample in MS, and 10,000 for MS/MS. PNAG oligomers were only found in the 25% (v/v) acetonitrile fractions.

### Crystallization, data collection, and structure solution

Purified *Bb*-GH was concentrated to 10 mg/ml and screened for crystallization conditions at 20°C using hanging-drop vapour diffusion in 48-well VDX plates (Hampton Research) with the MCSG 1–4 sparse matrix suites (Microlytic). An initial crystallization hit was obtained from MCSG-1 condition #77. Optimized crystals approximately 500 × 150 × 50 μM in dimension were grown using 8.6 mg/mL *Bb*-GH with a 3 μl drop with equal amounts of protein and precipitant equilibrated against 250 μl precipitant solution (BIS-TRIS pH 6.9, 0.2 M lithium sulfate, and 1.7 M ammonium sulfate) after one week of incubation. The crystals were cryoprotected for 5–10 s in reservoir solution supplemented with 25% (v/v) ethylene glycol prior to vitrification in liquid nitrogen. Diffraction data were collected at a wavelength of 1.075 Å on beam line X29A at the National Synchrotron Light Source (NSLS) (Table 2). The data were indexed, integrated, and scaled using HKL2000 [72]. The structure was determined by molecular replacement with PHENIX AutoMR [40] using *Ec*-GH (PDB 4P7L) [24] as a search model. The resulting electron density map enabled PHENIX AutoBuild [40] to build ~95% of the protein. The remaining residues were built manually in COOT [73] and alternated with refinement using PHENIX.REFINE [40]. Translation/Libration/Screw (TLS) groups were used during refinement and determined automatically using the TLSMD web server [74, 75]. Structure figures were generated using PYMOL Molecular Graphics System (DeLano Scientific, http://www.pymol.org/), quantitative electrostatics were calculated using PDB2PQR [76, 77] and APBS [78], and conservation levels were generated with ConSurf [79]. Programs used for crystallographic data processing and analysis were accessed through SBGrid [80].

## Supporting information

S1 Text Table A containing strains, plasmids, and primers used in this study. (DOCX)

S1 Fig Mono-deacetylated dPNAG oligomers contain a GlcN unit at site -3. (TIF)

S2 Fig Di-deacetylated PNAG oligomers contain GlcN units between sites -2 and -5. (TIF)

S3 Fig Size-exclusion chromatogram of *Bb*-GH suggests the protein is a monomer in solution. (TIF)

S4 Fig GlcNAc and GlcN monomer orientations are incompatible with polymer binding. (TIF)
