# Supplementary material for: PgaB orthologues contain a glycoside hydrolase domain that cleaves deacetylated poly-β(1,6)-N-acetylglucosamine and can disrupt bacterial biofilms
Source: PLoS Pathog. 2018 Apr 23;14(4):e1006998. doi: 10.1371/journal.ppat.1006998 (PMC5933820; doi:10.1371/journal.ppat.1006998)
Supplement: S1 Text — (DOCX) [file ppat.1006998.s001.docx]

**Supporting Information**

**PgaB orthologues contain a glycoside hydrolase domain that cleaves deacetylated poly-β(1,6)-*N*-acetylglucosamine and disrupt bacterial biofilms**

Dustin J. Little^1,2¶#^, Roland Pfoh^1¶^, François Le Mauff^3,4^, Natalie C. Bamford^1,2^, Christina Notte^1^, Perrin Baker^1^, Manita Guragain^5,6^, Howard Robinson^7^, Gerald B. Pier^8^, Mark Nitz^9^, Rajendar Deora^5,6^, Donald C. Sheppard^3,4^, P. Lynne Howell^1,2^*

^1^Program in Molecular Medicine, The Hospital for Sick Children, Toronto, ON, Canada

^2^Department of Biochemistry, University of Toronto, Toronto, ON, Canada

^3^Departments of Medicine and of Microbiology and Immunology, McGill University, Montréal, QC, Canada

^4^Infectious Diseases and Immunity in Global Health Program, Research Institute of the McGill University Health Centre, Montréal, QC, Canada

^5^Department of Microbiology and Immunology, Wake Forest School of Medicine, Winston-Salem, NC, USA

^6^Department of Microbial Infection and Immunity, The Ohio State University Wexner Medical Center, Columbus, OH, USA

^7^Photon Sciences Division, Brookhaven National Laboratory, Upton, NY, USA

^8^Division of Infectious Diseases, Department of Medicine, Brigham and Women's Hospital, Harvard Medical School, Boston, MA, USA

^9^Department of Chemistry, University of Toronto, Toronto, ON, Canada

**^#^** Current address: Department of Biochemistry and Biomedical Sciences, McMaster University, Hamilton, ON, Canada

*Corresponding author: howell@sickkids.ca (PLH)

^¶^These authors contributed equally to this work.

**Table A**: Strains, plasmids, and primers used in this study.

| Strain, plasmid, or primer | Description or characteristics | Source or reference |
| --- | --- | --- |
| Strains |  |  |
| TRXWMGΔABCD | *E. coli* K-12 MG1655 strain: F^-^ λ^-^ *csrA::kan* Δ*pgaABCD* | [34] |
| TOP10 | *E. coli* cloning strain: F^-^ *mcr*A Δ(*mrr-hsd*RMS-*mcr*BC) φ80*lac*ZΔM15 Δ*lac*X74 *rec*A1 *ara*D139 Δ(*ara-leu*) 7697 *gal*U *gal*K *rps*L (Str^r^) *end*A1 *nup*G λ- | Invitrogen |
| BL21 CodonPlus (DE3) | *E. coli* expression strain: F^-^ *omp*T *hsd*S(r_B_^-^ m_B_^-^) *dcm*^+^ Tet^r^ *gal* λ(DE3) *end*A [*arg*U *pro*L Cam^r^] | Stratagene |
| TM300 pTX15icaADBC | *S. carnosus* TM300 strain containing pTX15*icaADBC* | [35] |
| SE801 | *S. epidermidis* clinical isolate. | This study |
| Bp536 Δ*bps* | *B. pertussis* 536 strain with the *bpsABCD* locus deleted | [6] |
| DH5α | *E. coli* laboratory strain: F^-^ endA1 glnV44 thi-1 recA1 relA1 gyrA96 deoR nupG Φ80d*lacZ*ΔM15 Δ(*lacZYA-argF*)U169, hsdR17(r_K_^-^ m_K_^+^), λ– | Invitrogen |
| Plasmids |  |  |
| pPGA372 | *pgaABCD* in pUC19 | [34] |
| pMM11 | *B. bronchiseptica bpsABCD* operon cloned into pBBR1MCS | [12] |
| pETDuet-1 | Co-expression vector | Novagen |
| pET24a | Expression vector | Novagen |
| pET28a | Expression vector | Novagen |
| pETDuet-PgaCD | *E. coli* PgaC and PgaD co-expression plasmid | This study |
| pET24-WspR R242A | WspR R242A expression plasmid | This study |
| pET28-PgaB_22-672_ | *Ec*-DAGH expression plasmid | [67] |
| pET28-PgaB_310-672_ | *Ec*-GH expression plasmid | [24] |
| pET28-BpsB_27-701_-2 | *Bb*-DAGH expression plasmid C47S variant | [14] |
| pET28-BpsB_27-311_ | *Bb*-DA expression plasmid C47S variant | [14] |
| pET28-*Bb*-GH | *Bb*-GH expression plasmid | This study |
| pET28-*Bb*-GH D326A | *Bb*-GH D326A expression plasmid | This study |
| pET28-*Bb*-GH D326N | *Bb*-GH D326N expression plasmid | This study |
| pET28-*Bb*-GH D328A | *Bb*-GH D328A expression plasmid | This study |
| pET28-*Bb*-GH D328N | *Bb*-GH D328N expression plasmid | This study |
| pET28-*Bb*-GH Y329F | *Bb*-GH Y329F expression plasmid | This study |
| pET28-*Bb*-GH D364A | *Bb*-GH D364A expression plasmid | This study |
| pET28-*Bb*-GH D364N | *Bb*-GH D364N expression plasmid | This study |
| pET28-*Bb*-GH H473A | *Bb*-GH H473A expression plasmid | This study |
| pET28-*Bb*-GH D474A | *Bb*-GH D474A expression plasmid | This study |
| pET28-*Bb*-GH D474N | *Bb*-GH D474N expression plasmid | This study |
| pET28-*Bb*-GH Y549F | *Bb*-GH Y549F expression plasmid | This study |
| pET28-*Bb*-GH M584E | *Bb*-GH M584E expression plasmid | This study |
| pET28-*Bb*-GH E585A | *Bb*-GH E585A expression plasmid | This study |
| pET28-*Bb*-GH E585Q | *Bb*-GH E585Q expression plasmid | This study |
| pET28-*Bb*-GH Y648F | *Bb*-GH Y648F expression plasmid | This study |
| pET24a-DspB | DspB expression plasmid | This study |
| Primers |  |  |
| PgaC Fwd | GGGGGATCCGATTAATCGCATCGTATCG | This study |
| PgaC Rev | GGGAAGCTTTTAACCTCTCAGAATCCC | This study |
| PgaD Fwd | GGGCATATGAACAATTTAATTATTACG | This study |
| PgaD Rev | GGCTCGAGTTATGCCCGGACTAGCGC | This study |
| 318 Fwd | GGGCATATGCCGATCGAGCGCATCGTGCA | This study |
| 670 Rev | GGGAAGCTTCTACGACTTCAGCGACATCACATC | This study |
| D326A Fwd | GCGCATCGTGCAGGTCGCCCTGGACTAC | This study |
| D326A Rev | CGTAGATGTAGTCCAGGGCGACCTGCACG | This study |
| D326N Fwd | GCATCGTGCAGGTCAACCTGGACTACATC | This study |
| D326N Rev | GTCGTAGATGTAGTCCAGGTTGACCTGCACG | This study |
| D328A Fwd | CGTGCAGGTCGACCTGGCCTACATCTAC | This study |
| D328A Rev | CGGGGTCGTAGATGTAGGCCAGGTCGAC | This study |
| D328N Fwd | CGTGCAGGTCGACCTGAACTACATCTACGACC | This study |
| D328N Rev | GGTCGGGGTCGTAGATGTAGTTCAGGTCGACC | This study |
| Y329F Fwd | GTGCAGGTCGACCTGGACTTCATCTACGAC | This study |
| Y329F Rev | GGTCGGGGTCGTAGATGAAGTCCAGGTC | This study |
| D364A Fwd | CCTGCAGGCCTTCGCCGCTCCCAAGGGC | This study |
| D364A Rev | CGCCGTCGCCCTTGGGAGCGGCGAAGGC | This study |
| D364N Fwd | GCAGGCCTTCGCCAATCCCAAGGGCGACGG | This study |
| D364N Rev | GTCGCCGTCGCCCTTGGGATTGGCGAAGGC | This study |
| H473A Fwd | CGACGGCCTGCTGTTCGCCGACGACGCC | This study |
| H473A Rev | CGAGCACGGCGTCGTCGGCGAACAGCAG | This study |
| D474A Fwd | CGGCCTGCTGTTCCACGCCGACGCCGTG | This study |
| D474A Rev | CGTCGAGCACGGCGTCGGCGTGGAACAG | This study |
| D474N Fwd | GCCTGCTGTTCCACAACGACGCCGTGCTCG | This study |
| D474N Rev | GTCGTCGAGCACGGCGTCGTTGTGGAACAGC | This study |
| Y549F Fwd | GGTGGCGCGCAATCTCTTCGCCCAGCCG | This study |
| Y549F Rev | CGAGCACCGGCTGGGCGAAGAGATTGCG | This study |
| M584E Fwd | GATGGCCATGCCCAACGAGGAGGGCGCCGC | This study |
| M584E Rev | GCCGCGCGGCGCCCTCCTCGTTGGGCATGG | This study |
| E585A Fwd | GGCCATGCCCAACATGGCGGGCGCCGCG | This study |
| E585A Rev | CGGGCCGCGCGGCGCCCGCCATGTTGGG | This study |
| E585N Fwd | CCATGCCCAACATGCAGGGCGCCGCGCGGC | This study |
| E585N Rev | CTCGGGCCGCGCGGCGCCCTGCATGTTGGG | This study |
| Y648F Fwd | GCGCCATCAACTATGGCTACTTCCCGGACG | This study |
| Y648F Rev | GGCGATGAAATCGTCCGGGAAGTAGCCATAG | This study |


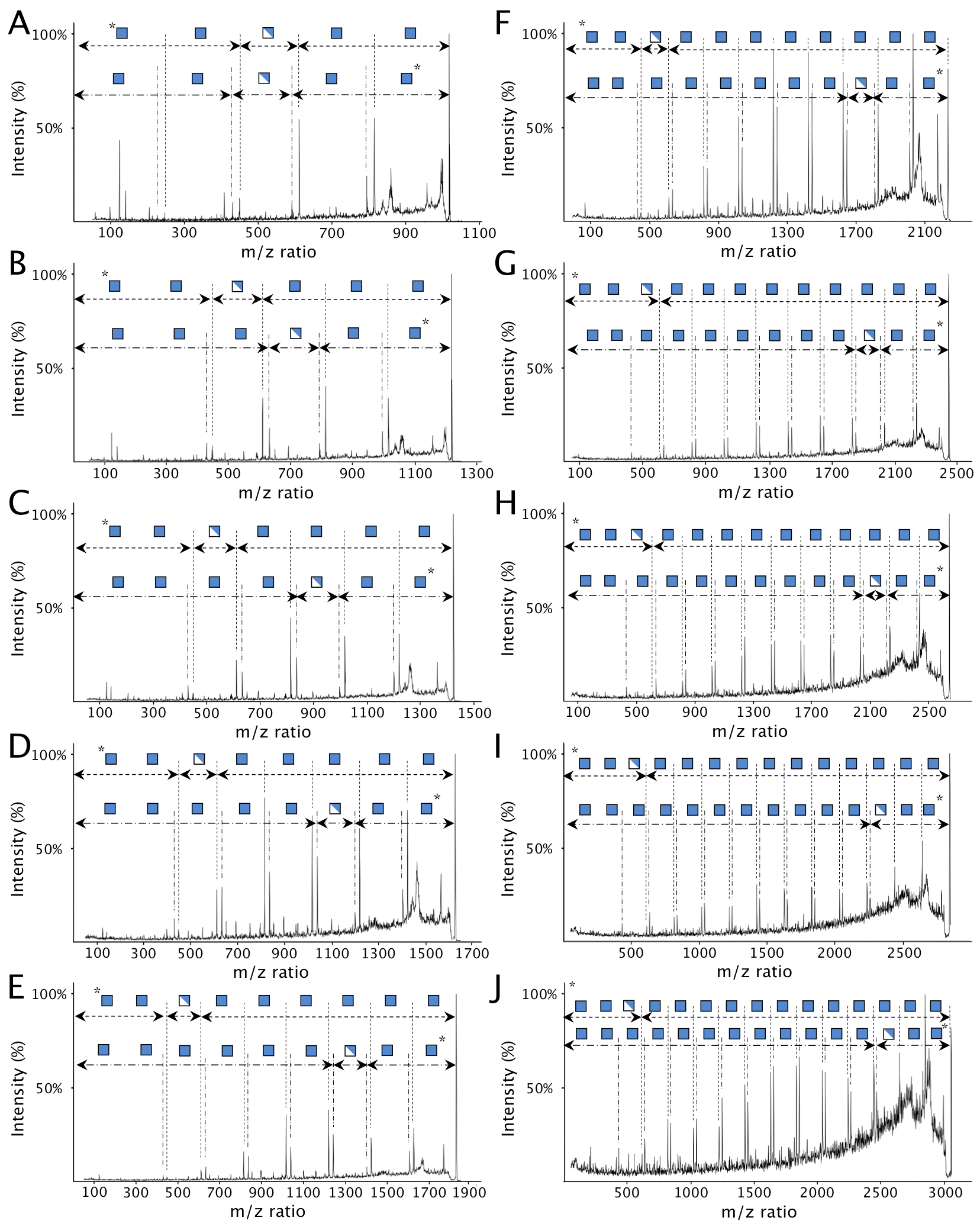


**S1 Fig.** **Mono-deacetylated dPNAG oligomers contain a GlcN unit at site -3.** MALDI-TOF MS/MS profile of the mono-deacetylated structures of pentasaccharide (degrees of polymerization five, DP5) (A), DP6 (B), DP7 (C), DP8 (D), DP9 (E), DP11 (F), DP12 (G), DP13 (H), DP14 (I) and DP15 (J). Blue square: GlcNAc, Half-blue square: GlcN, * denotes the reducing end of the polysaccharide.


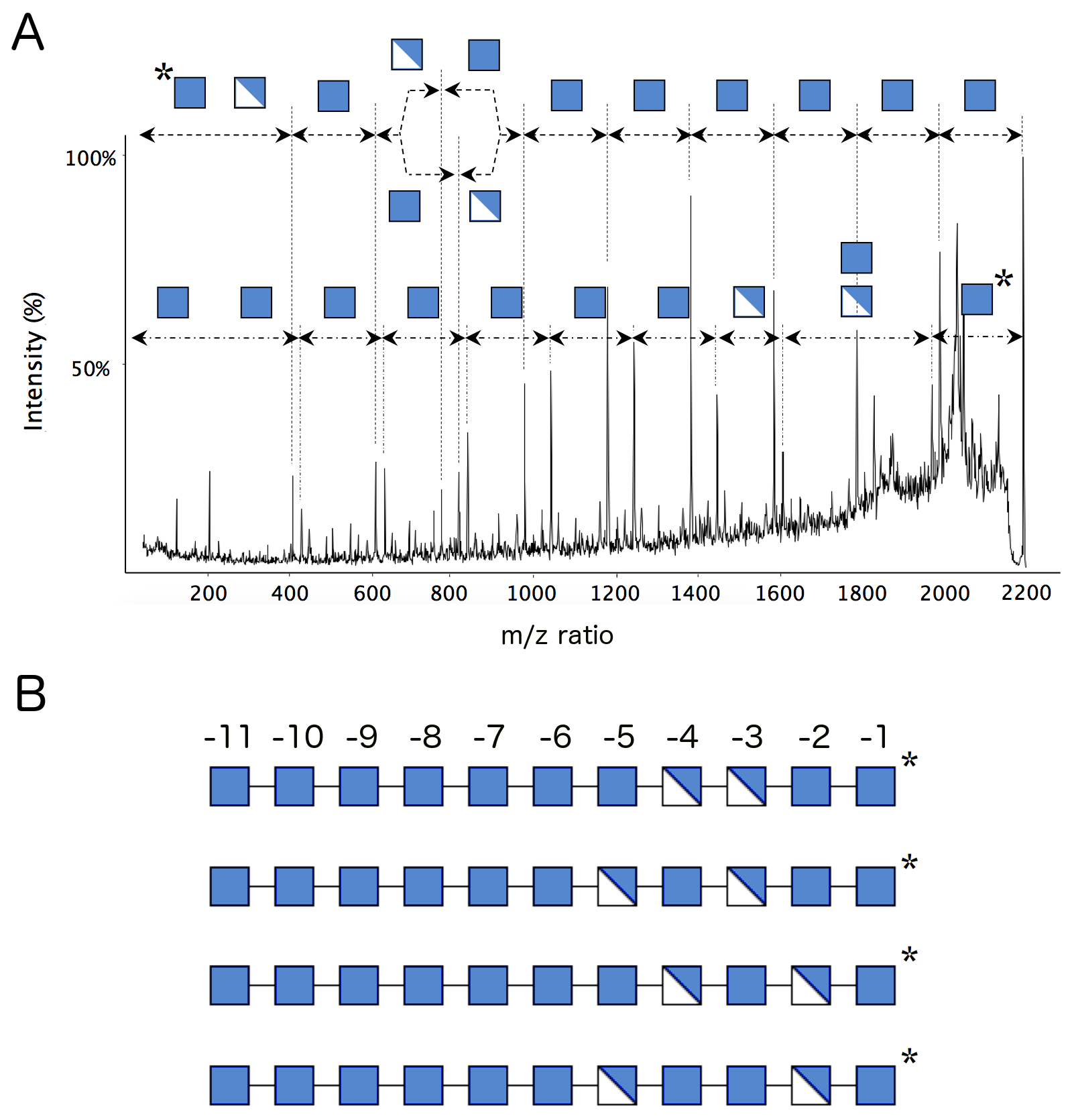


**S2 Fig.** **Di-deacetylated PNAG oligomers contain GlcN units between sites -2 and -5.** (A) MALDI-TOF MS/MS profile of the reduced di-deacetylated structure, GlcNAc_9_GlcN_2_, at m/z ratio 2192.85. (B) Graphical representation of the potential structures of the GlcNAc_9_GlcN_2_ produced by *Bb*-DAGH. Blue square: GlcNAc, Half-blue square: GlcN, * denotes the reducing end of the polysaccharide.


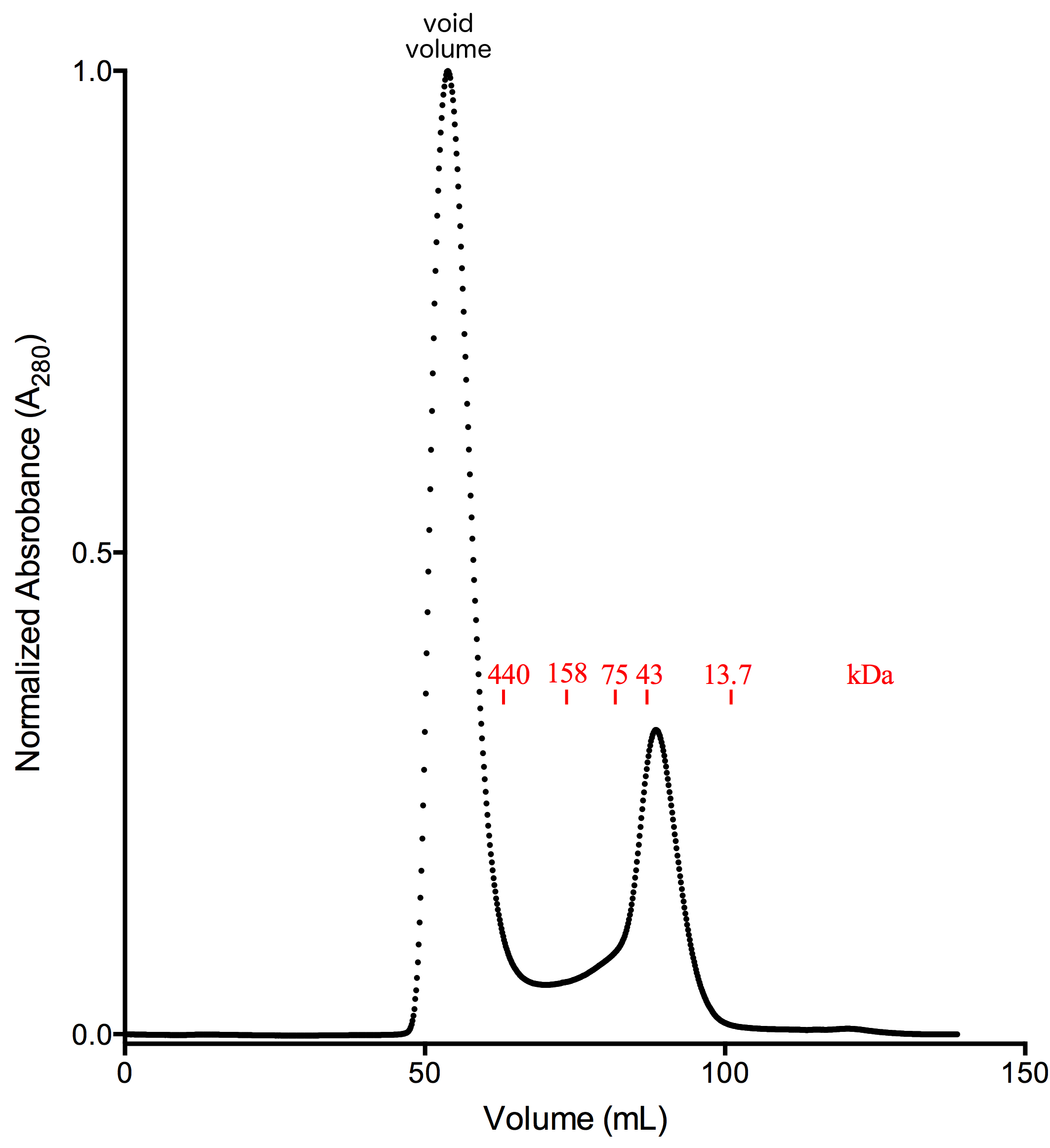


**S3 Fig. Size-exclusion chromatogram of *Bb*-GH suggests the protein is a monomer in solution.** Comparison *Bb*-GH to ferritin (440 kDa), aldolase (158 kDa), conalbumin (75), ovalbumin (43 kDa), and ribonuclease A (13.7 kDa) shows an elution profile corresponding to a molecular weight of ~40 kDa.


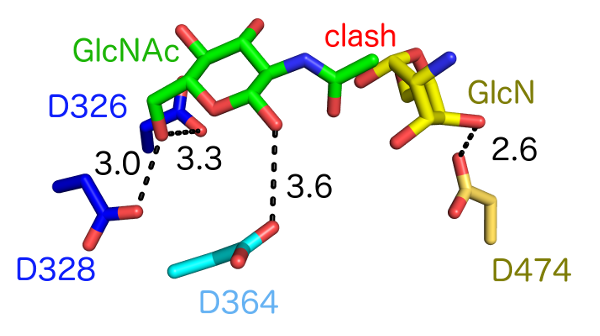


**S4 Fig. GlcNAc and GlcN monomer orientations are incompatible with polymer binding.** Shown in yellow and green sticks are the GlcN (PDB 4P7N) and GlcNAc (PDB ID 4P7Q) saccharides observed in *Ec*-GH [24], which were modeled into the *Bb*-GH structure. Note the clash between the acetyl-group of GlcNAc with GlcN. Dashed lines indicate hydrogen bonds to critical residues in the central pocket; bond lengths are given in Å.
