# Supplementary figures and images for: PgaB orthologues contain a glycoside hydrolase domain that cleaves deacetylated poly-β(1,6)-N-acetylglucosamine and can disrupt bacterial biofilms
Source: PLoS Pathog. 2018 Apr 23;14(4):e1006998. doi: 10.1371/journal.ppat.1006998 (PMC5933820; doi:10.1371/journal.ppat.1006998)

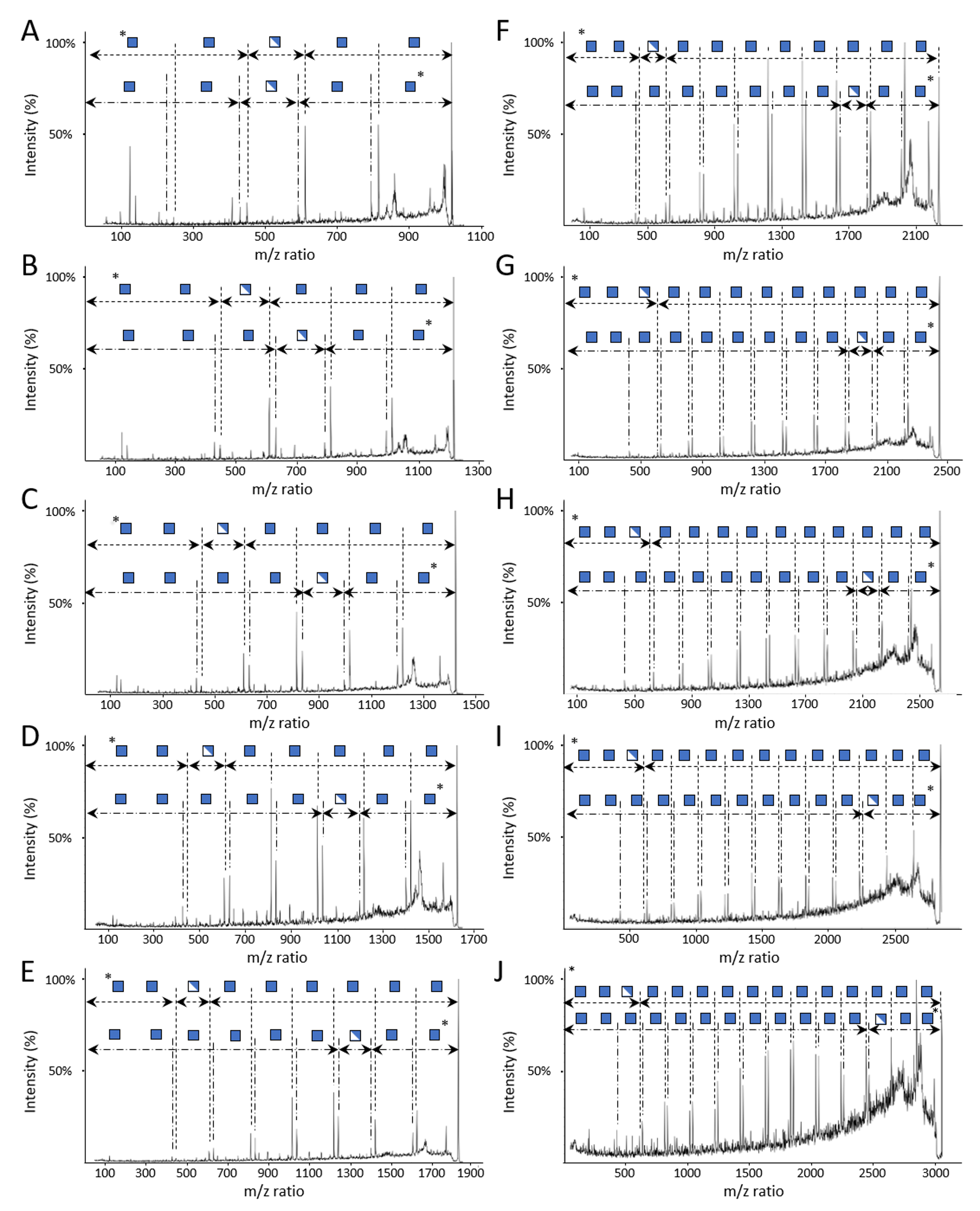

Supplement: S1 Fig — (TIF) [file ppat.1006998.s002.tif]

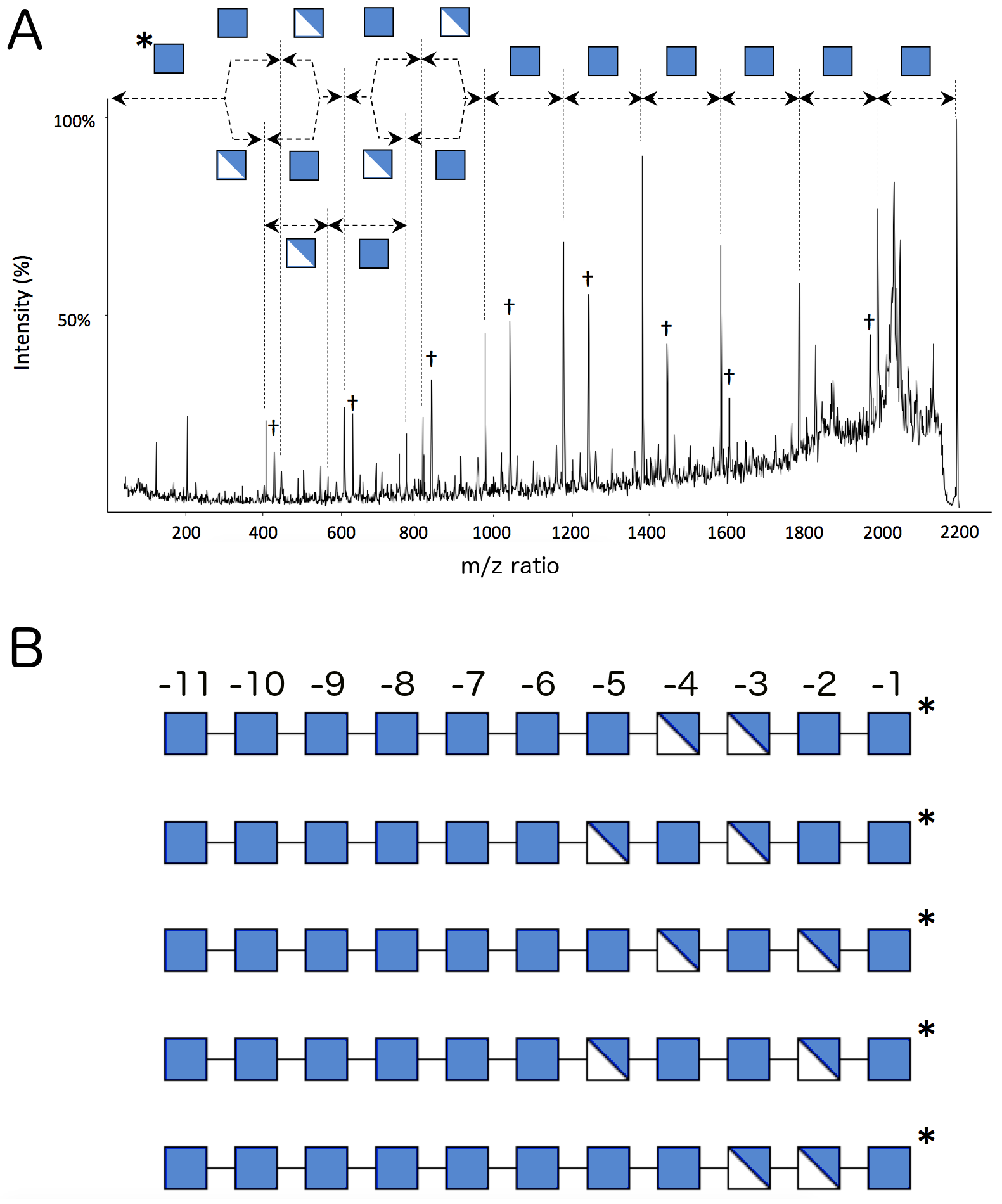

Supplement: S2 Fig — (TIF) [file ppat.1006998.s003.tif]

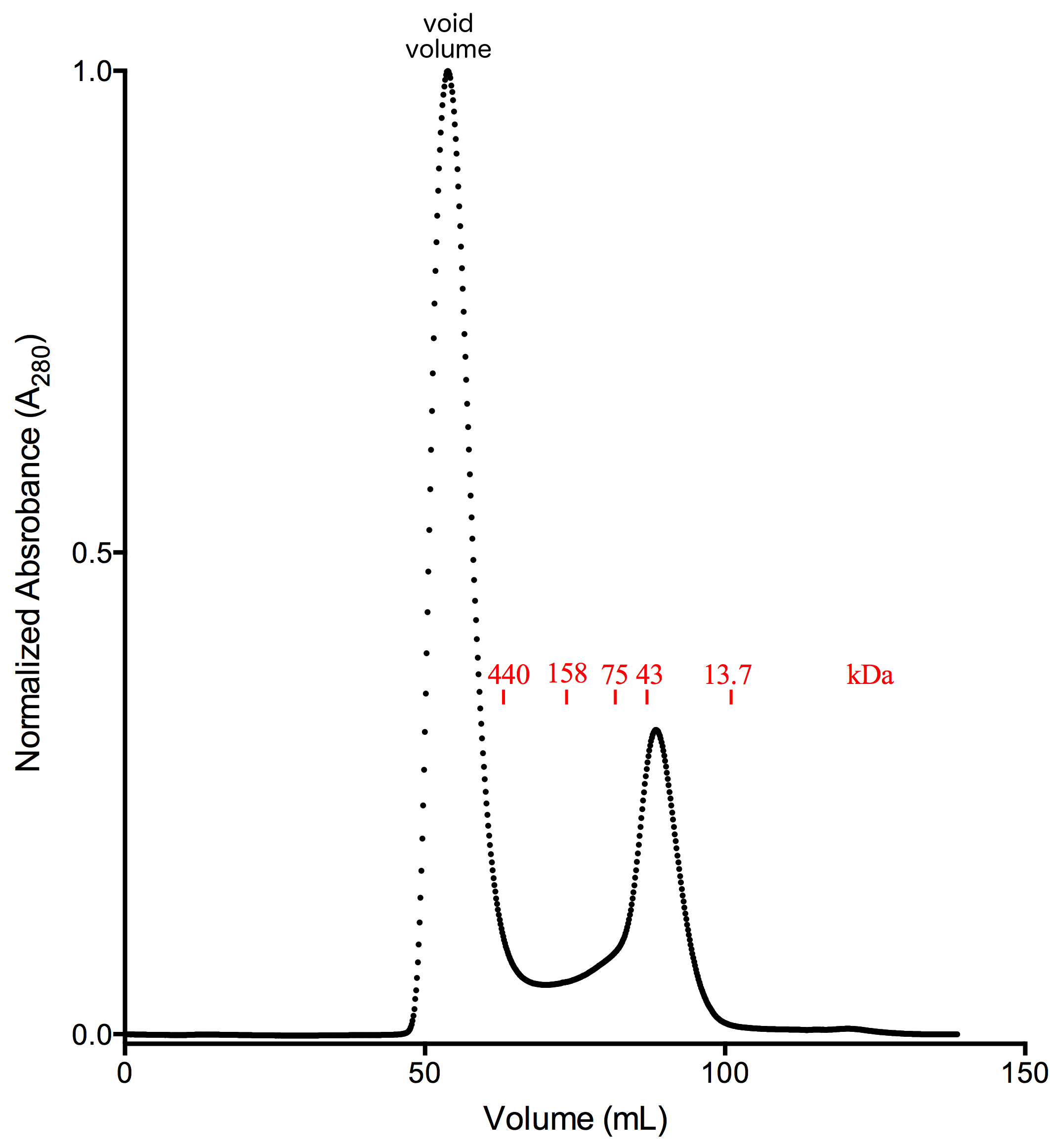

Supplement: S3 Fig — (TIF) [file ppat.1006998.s004.tif]

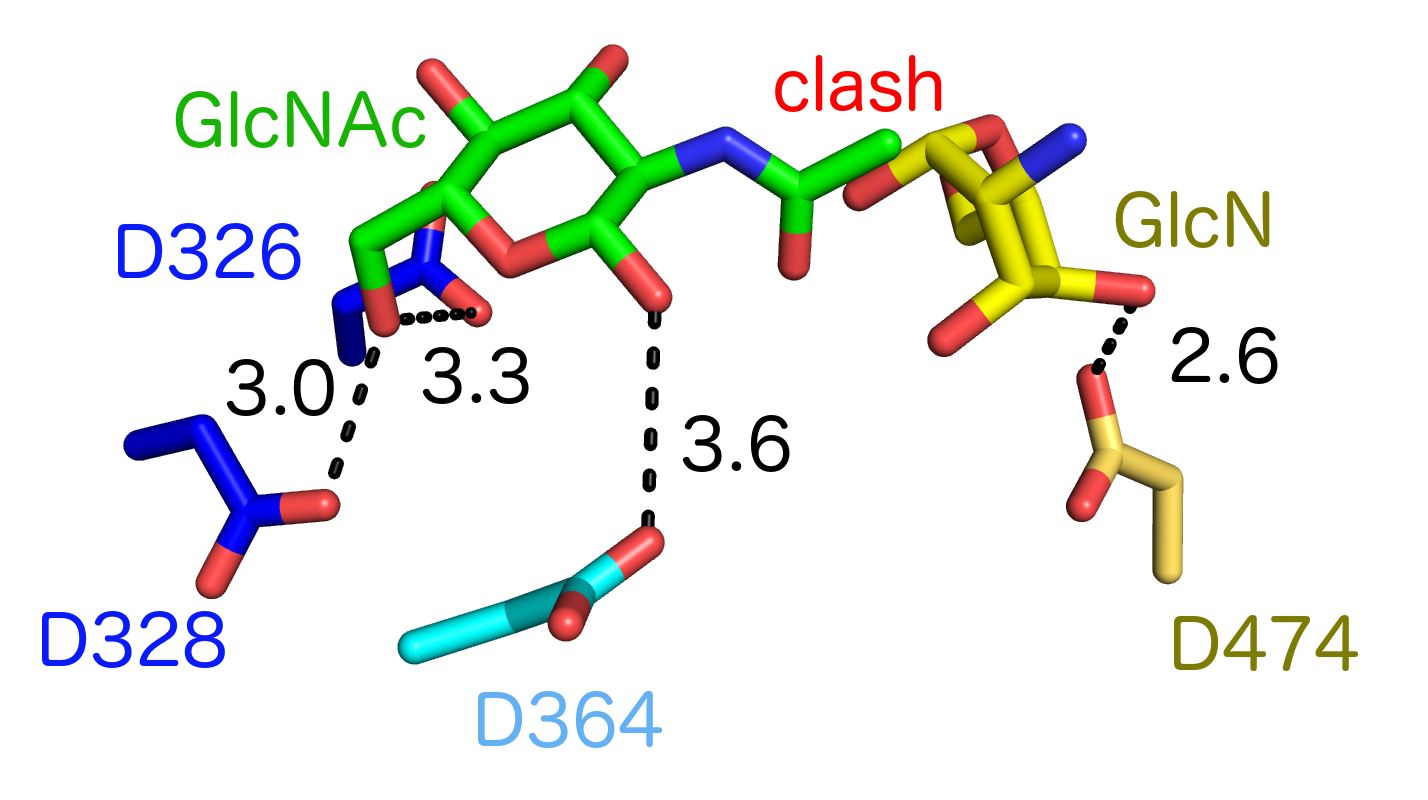

Supplement: S4 Fig — (TIF) [file ppat.1006998.s005.tif]
